# Effects of visual search training in children with hemianopia

**DOI:** 10.1371/journal.pone.0197285

**Published:** 2018-07-18

**Authors:** Iliya V. Ivanov, Stephan Kuester, Manfred MacKeben, Anna Krumm, Manja Haaga, Martin Staudt, Angelika Cordey, Claudia Gehrlich, Peter Martus, Susanne Trauzettel-Klosinski

**Affiliations:** 1 Vision Rehabilitation Research Unit, Centre for Ophthalmology, University of Tübingen, Tübingen, Germany; 2 ZEISS Vision Science Lab, Institute for Ophthalmic Research, Centre for Ophthalmology, University of Tübingen, Tübingen, Germany; 3 The Smith-Kettlewell Eye Research Institute, San Francisco, California, United States of America; 4 Pediatric Neurology, University Children’s Hospital Tübingen, Tübingen, Germany; 5 Schön Klinik Vogtareuth, Clinic for Neuropediatrics and Neurorehabilitation, Vogtareuth, Germany; 6 Institute for Clinical Epidemiology and Applied Biometry, University of Tübingen, Tübingen, Germany; Universitat Regensburg, GERMANY

## Abstract

**Background:**

This study investigates the effect of a new computer-based visual search training (VST) that was adapted for children with homonymous hemianopia (HH).

**Methods:**

22 children with HH (median age 11 years, 8 months: 6y6m-19y2m) trained at home for 15 minutes twice/day, 5 days/week, for 6 weeks. To assess performance before training (T1), directly after training (T2) and 6 weeks after the end of training (T3), we measured search times (STs) during on-screen search (with eye tracking), and in a real life search task. Additional variables analyzed during on-screen search were numbers, amplitudes, and durations of saccades, their directional patterns and the proportional number of saccades into the non-seeing field. The latter was the main variable during free viewing. Sixteen healthy age-matched children, who did not undergo the training, served as comparison group. Quality of Life (QoL)-questionnaires were also applied.

**Results:**

STs of the patients decreased significantly during the training and all search performance tests. This improvement persisted 6 weeks after the end of the training. Saccade amplitudes increased, total number of saccades to find the target decreased, and the proportional number of saccades to the non-seeing side increased. These changes were maintained at T3. Saccade durations did not change. During free viewing, saccades were equally distributed to both sides before and after training. Patients reported improvements in QoL and activities of daily living. Performance in the healthy children did not change by simply repeating the visual search test.

**Conclusions:**

The improvement in STs in all search tasks, larger and fewer saccades, and an improved search strategy after VST suggests that the children with HH benefited from the training. The maintained improvement at T3 and the improvement in the real life search task indicate that the newly developed search strategy persists and can be applied to everyday life.

## Introduction

Homonymous hemianopia (HH) causes impaired spatial orientation and diminished quality of life [1,2]. Much research exists on adult patients with HH [3–8], but only few studies on children. To our knowledge, the present study is the first using visual search training (VST), eye movements and exploration of relevance for daily living in children with HH. Several aspects are especially interesting in children: What is the best way to assess the visual fields? Do children with HH adapt spontaneously? Do adaptations and the effect of training depend on disease duration or age at examinations? Is head posture before training a measure of spontaneous adaptation?

Regarding the term adaptation, we will use “spontaneous adaptation” as a general term for a reaction to the visual impairment, “spontaneous maladaptation” for a functionally ineffective or negative reaction to the visual impairment. “Spontaneous” means “without intervention”, “natural development” describes the improvement of functions through the natural aging process (in healthy and impaired children).

Hence, this study of children with HH focused on 3 main questions:

1. Is there evidence for spontaneous adaptations at baseline before training?
2. What were the effects of VST?
3. What were the differences compared with healthy children?

The experimental paradigm consisted of three different search tasks. We investigated whether children can benefit from them regarding the following points:

- Improvement of STs through repetition during training,
- Improvement of STs in on-screen search tasks during eye tracking, and
- Impact on daily living through solving a real life search task.

In addition, we asked the participants for a self-assessment through questionnaires.

It is well known that spontaneous adaptations in adults can be supported by compensatory training [9–12] (for reviews see [3–8,13]). We had shown the effectiveness of VST in adult patients with HH in a randomized, controlled study [2]. In a case series including three teenagers with homonymous visual field loss it has been reported that audio-visual compensatory training may improve the visual response [14]. Perceptual training that combined attentional tracking with a spatially and temporally unpredictable motion discrimination task has been effective in training of visually impaired children that exhibit decreased peripheral perception even in absence of obvious peripheral deficits [15]. As these children represented a heterogeneous group of children with low vision (visual acuity 20/200 or less) and all had eye diseases, they are not directly comparable to those with a suprachiasmal lesion. Furthermore, possible effects of restitution therapy in the lost visual field of children with HH were investigated [16,17]. However, when fixation control is insufficient during visual field testing, improvement of visual fields can be mimicked by a shift of the visual field border due to eye movements [18]. At present, only studies using compensatory methods in adults are evidence-based (for reviews see [3,6,8,13]).

Only few studies on brain damage in children [19–22] have focused on HH. Some of them dealt with visual field examination [23,24] and with adaptation [23,25–28], which was reported to be better in congenital vs. acquired brain damage [29].

Eye movements in children with HH were recorded in a study using a single saccadic task [30]. Regarding earlier training methods, it has been emphasized that the training task needs to be adapted to everyday demands [31].

## Methods

### Study design

We realize that the optimal study design should include a control group of patients with HH who do not receive treatment or a placebo training. This group should ideally be chosen by random selection. There were three reasons why we felt that we needed to choose a different solution for the study design:

1. We had to acknowledge that HH without major cognitive deficits and additional signs of cerebral visual impairment [19–22] is a rare condition in children, so that the availability of participants for our study would be low. Thus, by cutting the available cohort in half to create proper experimental and comparison groups would have prolonged the study unacceptably.
2. Because of the rarity of the condition, we knew we would have to include children who might live far away from our clinic. Most children with HH have serious mobility problems and are in no condition to travel such distances alone. Thus, the necessary efforts to implement a study in which half the participants would not have a chance to benefit from the treatment would have been wasteful. Note that the option of a cross-over design would have necessitated more visits for the children, which would have been unrealistic due to limited ability to travel.
3. Most parents knew that we had shown in a previous study that a training program was successful in adult patients. This is why many of them assumed that the same procedure could help their children as well. Thus, we knew it would have been hard to get them to accept that their child might end up in a control group without treatment.

For these reasons, we decided to give all affected children the same chance to participate in a study that could potentially benefit them.

In addition, we also wanted to find out to which level of performance the patients could aspire at the end of the training. This is the reason why we also measured search performance in a comparison group of healthy, age-matched children who were not suffering from HH. The goal was to see whether hemianopic children can reach normal performance levels. Training the healthy children was not an option, because starting with normal STs they would have reached a floor effect quickly. In contrast to the hemianopic children, the healthy children did not have a good enough reason to stay motivated to perform the training task, so that not much compliance could be expected. Instead, the healthy children were assigned to do a 15 minutes’ test-session—a single session from the training program of the patients—at baseline and 6 weeks later–to examine whether they improve just by repeating the task.

**Fig 1:**
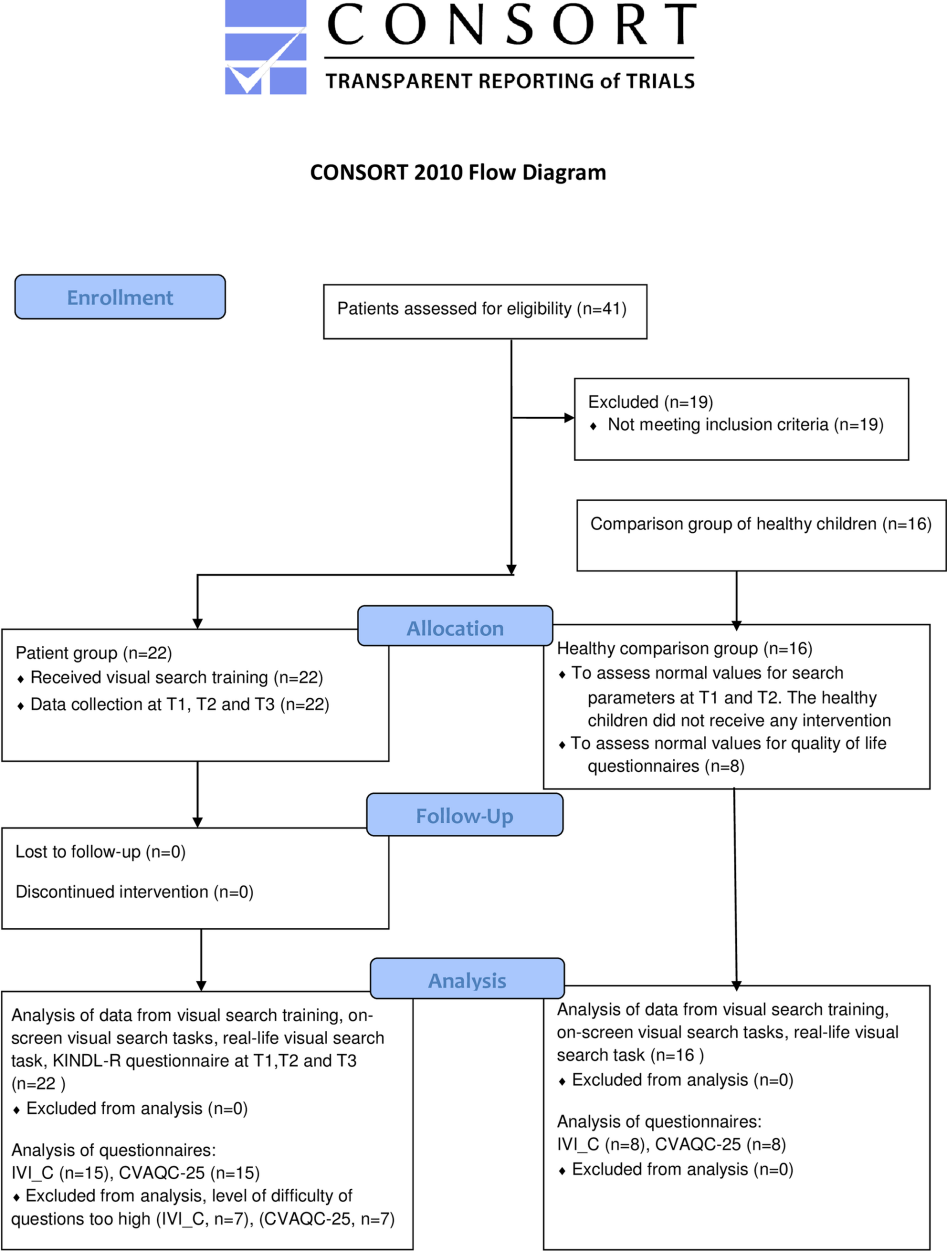
Flow diagram of the study.

A flow chart of the study design is shown in Fig 1.

### Participants

Twenty-two children (10 males) with focal brain lesions and HH (13 right, 9 left, 3 quadrantanopias) were included (see Table 1).

**Table 1:** Demographic and clinical data.

Patient ID	Age (y;m)	Age at onset (y;m)	Disease duration (y;m)	Sex	Cause of HH	Visual field	Epilepsy	Hemiparesis	Head turn ipsilateral	Exotropia ipsilateral	Eccentric fixation
1	6;6	1;9	4;10	F	hemiatrophy	HH r	+	+	+	-	n.a.
3	14;6	perinatal	14;6	F	head injury	HH r	+	+	+	+	n.a.
4	9;7	9;5	0;2	M	resection of FCD	Q upper r	-	-	-	-	n.a.
6	7;11	perinatal	7;11	M	perinatal ischemia	HH r	-	+	+	-	+
7	12;3	perinatal	12;3	M	perinatal ischemia	HH r	+	+	+	+	+
8	10;1	4;2	5;11	F	resection of FCD	HH r	-	+	+	-	+
9	16;8	perinatal	16;8	F	perinatal ischemia	HH l	+	+	+	+	+
10	16;10	16;1	0;9	M	head injury	HH l	-	+	+	-	-
11	13;7	5;8	7;11	F	hemispherotomy	HH r	-	+	+	-	+
12	16;9	5;6	11;3	M	hemiatrophy	HH l	-	+	+	-	-
13	7;3	0;11	6;4	F	tumor resection	HH r	+	+	+	+	-
14	11;11	10;0	1;11	M	tumor resection	Q upper r	+	-	-	-	-
15	14;1	9;3	4;10	M	hemorrhagic stroke	Q lower l	-	+	+	-	-
16	11;5	7;2	4;3	M	hemorrhagic stroke	HH l	-	+	+	-	-
17	10;5	2;9	7;8	F	hemispherotomy	HH l	-	+	+	+	-
19	11;4	perinatal	11;4	F	perinatal ischemia	HH r	+	-	+	+	-
20	17;10	9;6	8;4	F	tumor resection	HH l	-	+	+	-	-
25	7;7	0;9	6;10	F	ischemic stroke	HH l	-	+	+	-	+
28	12;7	6;8	5;11	M	tumor resection	HH r	-	-	-	-	-
22	7;6	7;3	0,3	M	hemispherotomy	HH l	+	+	+	-	-
31	9;9	9;8	0,1	F	tumor resection	HH r	+	-	+	+	-
30	19;2	0;1	19;1	F	hemispherotomy	HH r	+	+	+	-	-
**Median**	11;8	4;10	6;7								
**IQR**	5;10.5	9;2.75	7;7.25								
**Min**	6;6	perinatal	0;1								
**Max**	19;2	16;1	19;1								

age = age at first examination (T1), FCD = focal cortical dysplasia; F = female; HH = homonymous hemianopia; l = left; M = male; m = month; n.a. = not available; Q = quadrantanopia; r = right; y = year; ‘+' = yes; ‘-' = no; IQR = Interquartile Range.

Inclusion criteria were: Age 6–20 years, definitive diagnosis of HH or quadrantanopia, brain lesion diagnosed by MRI. The MRI findings are reported in a separate study [32].

Exclusion criteria were: Eye disease and/or additional field defects. In a few cases we were compelled to exclude a severely handicapped child due to insufficient cooperation during the first clinical examination.

The date range for participant recruitment and follow-up was October 22th 2014 through January 25th 2016. They were recruited from the Pediatric Neurology, University Children’s Hospital, Tuebingen, Germany and the Schön Klinik Vogtareuth, Clinic for Neuropediatrics and Neurorehabilitation, Vogtareuth, Germany.

The median age was 11 years, 8 months (6y6m -19y2m). The onset of the disease varied between congenital (5/22) and later acquired HH (17/22, median 6y8m, 0y1m-16y1m). Causes of HH were ischemia (n = 5), tumor (n = 5), epilepsy surgery (n = 6), trauma (n = 2), cerebral hemorrhage (n = 2) and other causes (n = 2). Ten of the 22 children suffered from epilepsy and 17 from hemiparesis.

Sixteen healthy, normally sighted children served as an age-matched comparison group (median age 11y6m: 7y8m- 17y3m).

The study was approved by the ethics committee of the University of Tuebingen Medical Faculty, and informed written consent was obtained from parents and children. The research adhered to the tenets of the Declaration of Helsinki.

### Neuro-ophthalmologic and orthoptic examination

All children underwent complete neuro-ophthalmologic and orthoptic examinations, which included best-corrected visual acuity (far/near), binocular status, motility, head posture, eye morphology, and fixation locus by direct ophthalmoscopy to assess potential homonymous eccentric fixation.

Visual fields were examined to document the HH, but the choice of the method was dependent on the cooperation of the individual. If possible, 30° kinetic perimetry by Tuebingen Manual Perimeter (Oculus, Wetzlar, Germany) was performed on both eyes. Otherwise, we examined binocular fields by tangent screen, confrontational perimetry (stimulus approaching from behind the child) or by a custom campimeter. This semi-quantitative method assessed eye movements towards the stimulus, presented as a LED light point on a semi-opaque screen, while the examiner observed the child’s gaze from behind the screen. Behavioral visual field screening [23,33] and gaze field assessment [24] have been reported earlier to be a promising methods in children.

### Performance

Visual search (VS) was used as a paradigm to collect behavioral data, with ST as the main outcome variable.

Performance was measured in three ways:

1. On-screen VS testing during eye tracking: ST while viewing high resolution photographs on a computer screen.
  Additional parameters tested: total number of saccades, their amplitudes and durations, and the relative number of saccades towards the non-seeing side.
2. On-screen free viewing testing during eye tracking, with the relative number of saccades towards the non-seeing side as the main variable.
3. Real life VS testing by the table test (a search task in a natural environment), with ST as the main variable.

In addition, QoL and mobility questionnaires were applied.

The testing schedule was set to assess a) performance at baseline, i.e. before training (T1), b) a potential effect after training (T2), and c) potential maintenance 6 weeks after the end of training (T3).

An overview of the study schedule and the examinations is given in Table 2.

**Table 2:** Study schedule.

examinations	T1(pre-training)	visual search training at home	T2 (post-training)	Pause no training	T3 (follow-up)
entry examination	**+**				
on-screen visual search testing	**+**		**+**		**+**
Real life visual search testing	**+**		**+**		**+**
quality of life and mobility questionnaires	**+**		**+**		**+**
time		6 weeks		6 weeks	

### Visual search training

**Fig 2:**
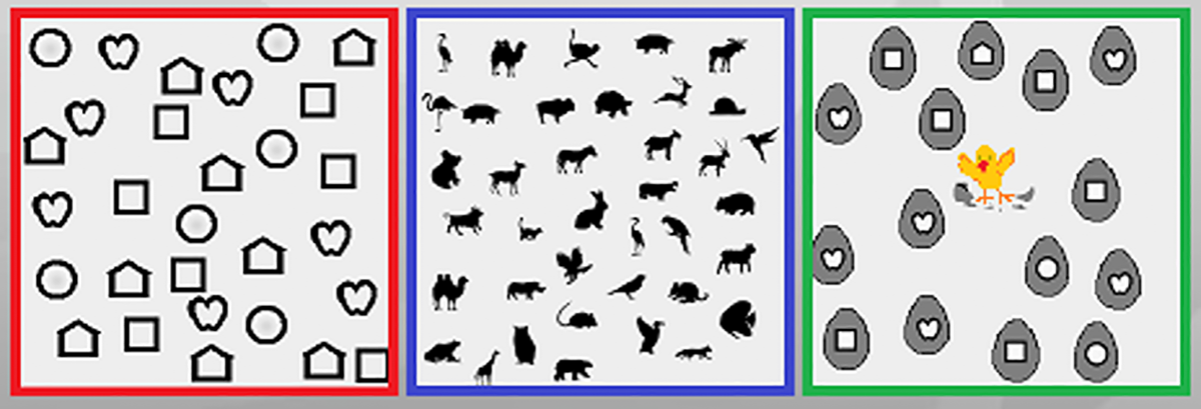
Search targets of the three levels of difficulty. Lea symbols at level 1 (left), animal silhouettes at level 2 (middle) and small Lea symbols on eggs at level 3 (right).

Based on a training method that we had used on adults [2], we modified the computer-based VST especially for children. The subjects performed a search task on a screen showing targets (symbols, 1.5–2° in size). The program was adapted for children on three levels of difficulty by using different sets of targets: The levels differed in the variety and complexity of the targets and distractors (see Fig 2).

At level one we used simple shapes (Lea symbols) for the search targets and distractors. At level two we used animal silhouettes, while at level three we chose the same shapes as at level one, but smaller and surrounded by a gray border (“animals hatching from eggs”). It turned out that level 2 was the most difficult level, where the children needed the longest STs.

Six targets and 34 distractors were distributed randomly on each screen, equalizing the location of appearance in all four quadrants over the entire session. The children had to find the six targets on each search screen and click on them using the computer mouse. All children were able to control the computer mouse, including those with hemiparesis.

The children were instructed to avoid head movements while searching for the targets. The viewing distance was adapted to the screen size of the child's computer to obtain a screen size of 60° horizontally and 40° vertically.

The children with HH trained at home for six weeks (15 min twice a day, five days/week) at levels of difficulty one, two, and three consecutively for two weeks each.

Training data were collected automatically and stored for later analysis.

### On-screen visual search testing with eye movement recordings

#### Apparatus

Eye position was sampled at 1000 Hz with an eye tracking system that utilizes infrared technology and is optimized for easy set-up and minimal intrusiveness (JAZZ-novo, Ober Consulting, Poznan, Poland).

The experiment was controlled by custom software. Viewing was binocular, while the system allowed tracking only the right eye. Saccades and fixations were detected using functions for detecting eye fixations in raw eye tracking data. Saccade detection was performed using a velocity-based algorithm proposed by Engbert and Kliegl (2003) [34].

The algorithm labels segments as saccades if the velocity of the eye movement exceeds a velocity threshold. The computation of velocity thresholds for the detection algorithm is based on the median of the velocity time series to protect the analysis from noise. A multiple (λ = 6) of the standard deviation of the velocity distribution is used as the detection threshold. We did not discriminate between microsaccades and large saccades. We analyzed all saccades detected by the algorithm with amplitudes larger than 0.2 degrees, twice the minimal resolution of the tracking device. Images were presented on a 21-inch gamma-corrected CRT monitor with a refresh rate of 120 Hz.

#### Stimuli

Subjects were presented full color 1280x1024 pixel photographs of real-world scenes (subtending a visual angle of 60° horizontally and 40° vertically) depicting a variety of everyday life outdoor scenes. Visual clutter in images of these scenes may vary greatly, which can influence eye movement strategies [35]. Image clutter was estimated by calculating measures like feature congestion, sub-band entropy and edge density, as proposed by Rosenholtz et al. (2007) [35]. Gabor patches were used as targets to ensure that their location could not be predicted by local or global scene content.

#### Procedure

**Fig 3:**
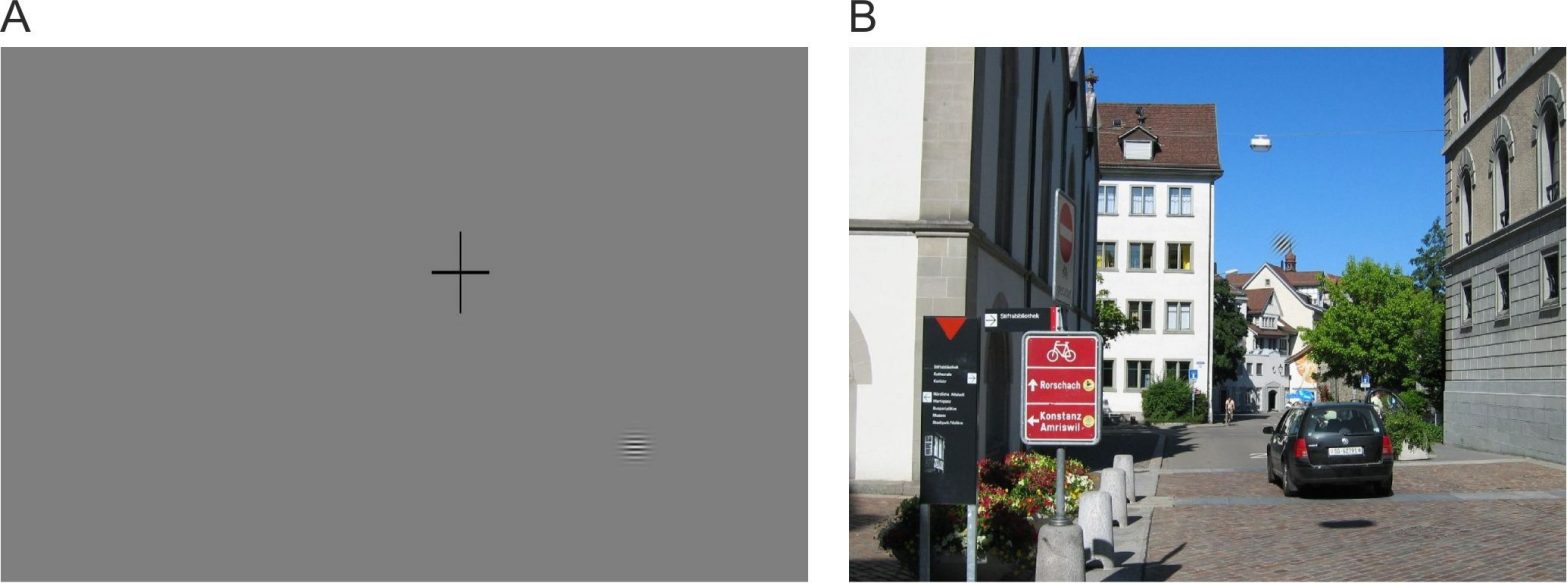
Schematic representation of the on-screen visual search task during eye tracking. A: Before each trial, a central cross was presented on a gray background to ensure the subject’s fixation at the center of the screen. A Gabor patch was displayed as an example of the search target: The instruction was “Find the zebra stripes”. B: The children were asked to search the images for the Gabor patch as quickly as possible (here on top of the chimney), and to press the space bar on the keyboard when they found it.

In the VS task, subjects were instructed to search for a superimposed Gabor patch in a set of 16 images as quickly as possible (Fig 3A and 3B). Each Gabor patch had a spatial frequency of 8 cpd and a radius of 1.7 deg at half maximum amplitude, presented at 100% luminance contrast, and their placement was pseudo-random. This resulted in 16 different image presentations per session. The same image sets were used at the pre-training (T1) and follow-up tests (T3), while a different one was selected at T2 in order to prevent possible bias in search behavior due to familiarity with the scenes. The target position varied randomly within each clutter area in each trial.

Before each trial, a central fixation cross was presented on a gray background to ensure the observer’s fixation on the center of the screen. The trial started automatically and a random image from the VS set was selected. The children indicated that the target had been found by pressing the space key. In the event that the target was not found, which was considered as an error, the trial timed out after 60 s and a new image was presented. For such trials, a ST of 60 s was stored in the data set. Before beginning the session, the children were shown examples of the search target, and the trial only began when observers understood the VS task (see Fig 3).

In the Free Viewing (FV) task, observers fixated a central cross on a gray background and initiated the trial by pressing the space key of a keyboard. Then a single image with medium complexity was presented for 10 seconds on the screen. Eye position recording started with the image presentation and was terminated as soon as the image presentation time of 10 s expired (measured with millisecond precision). The children were instructed to “just look at the image” and were not given specific instructions to attend to particular image regions or to find a target on the screen.

The following eye movement parameters were calculated and analyzed for each trial from the eye position recordings: Total number of saccades; saccade amplitude; average duration of saccades; mean number of saccades to the seeing and non-seeing hemifields.

Eye position recordings were analyzed separately for the VS and FV tasks. Each saccade that brings the fovea to the point of interest is preceded and followed by a fixation period. Thus, the conditions that determine which position of the monitor screen is visible changes with every saccade that the patient performs. Therefore, to count the number of saccades directed into the seeing or non-seeing hemifield, we analyzed each saccade direction dynamically relative to its prior location of fixation. Furthermore, for this analysis, we counted only saccades with a horizontal component exceeding its vertical component.

### Relevance for daily living

#### Real life visual search task: The table test

This semi-quantitative method analyzes search performance in a natural environment [2] and is an indicator of performance in daily living. 16 familiar real objects (4/quadrant) were placed in the 30° visual field on a table in front of the child. The examiner held up a duplicate object, and the children were asked to find the same object on the table by only using eye movements and then point at the object with their index finger. An assistant gently held the head steady to minimize head movements. ST was measured in seconds/object, and the sum of STs for all objects was used for further calculations.

#### Questionnaires

We used three questionnaires for children to assess QoL:

The Health-Related Quality of Life (HRQoL) KINDL® questionnaire [36] considers six domains that include physical and emotional well-being, self-esteem, family, friends and school. A global HRQoL score can be calculated after transforming the raw data on a scale ranging from 0 to 100, with higher scores indicating better HRQoL. All 22 children (n = 15 of 13 years or below, n = 7 of above 13 years) and their parents answered the questions.

For the KINDL questionnaire, a reference group was already available from the BELLA study ("**BE**fragung zum see**L**ischen Woh**L**befinden und Verh**A**lten“), which is the module on mental health and health-related quality of life within the German Health Interview and Examination Survey for Children and Adolescents (KiGGS) [36].This survey included 2863 parents of healthy children and adolescents 7–17 years of age and 1700 healthy children aged 11–17 years, who all completed the KINDL® quality of life questionnaire.

The second questionnaire was the Vision Impairment questionnaire for Children (IVI_C) [37]. It assesses vision-specific restrictions of participation in daily life. It has 24 Likert-scaled items, divided into 4 domains: Mobility (8 items), Social interaction (8 items), School (5 items) and Emotions (3 items). There were 5 response categories: “never”, “almost never”, “sometimes”, “almost always”, “always” and “no for other reasons”. All items answered with “no for other reasons” were declared as missing variables and excluded from the analysis. For each domain the median was calculated and transformed on a scale from 0–100, where higher scores indicate a higher QoL. Only a subgroup of 15 patients and 8 healthy children answered the IVI-C questions due to the difficulty of the questions.

The third questionnaire was the 25-item Cardiff Visual Ability Questionnaire for Children (CVAQC- 25) that probes vision-specific activities of daily life [38]. It has 25 Likert-scaled items with 7 domains: “Education”, “Near vision”, “Distance vision”, “Getting around”, “Social interaction”, “Entertainment” and “Sports”. All items answered with “don’t do for other reason/not interested in doing this” were considered as missing data and excluded from the analysis. A subgroup of 15 patients and 8 healthy children answered the CVAQC-25 questionnaire. We calculated the median for each domain.

As the IVI-C and the CVAQC-25 questionnaires were only available in English, we had to translate them into German.

In addition to the three questionnaires to assess the children’s QoL, we asked two specific questions to get additional information about difficulties regarding mobility that children with HH are facing:

“Do you/Does your child have difficulties getting out of the way of objects, vehicles or people?”

and

“Do you/Does your child have difficulties bumping into objects?”

Possible answers were “yes” or "no”. 18 children and their parents answered the specific mobility questions.

### Study registration

The study was reported to an open-source online registry as a non-randomized interventional study (No. DRKS00012695), German Clinical Trials Register, Cologne, Germany. The authors confirm that all ongoing and related trials for this intervention are registered. Regarding the definition of a clinical trial, the classification of our study was not based on the definition used by the WHO but rather on the guidelines for good clinical practice (ICH-GCP of July 2002, paragraph 1.12).

We digressed from the original study protocol by extending the maximum age for inclusion from 18 to 20 years, if the development of the child was slightly retarded by clinical examination.

A flow chart of the study is presented in Fig 1.

### Statistical methods

In all tests, α = 0.05 was accepted as a level of significance. Unless otherwise stated, the Shapiro-Wilk normality test and graphical Q-Q plots were used to test the shapes of different distributions. All eye movement data were normally distributed except the total number of saccades performed by the patients at T1. The training data were normally distributed after reciprocal transformation. Real life VS and self-reported data were not normally distributed.

#### Training

The STs during the training were positively skewed at the beginning and end of training at each level of difficulty. Thus, we performed a reciprocal transformation on the STs $\left(\frac{1}{search\;time}\right)$ to obtain normally distributed data. The reciprocal STs were confirmed to be normally distributed. All confirmatory statistical analyses were carried out on the transformed data. We show boxplots and report descriptive data by back-transformed original data. Relative improvement of ST was defined as $\left(\frac{{ST}_{T1}-{ST}_{T2}}{{ST}_{T1}}\right)$ if large values are unfavorable and as $\left(\frac{{ST}_{T2}-{ST}_{T1}}{{ST}_{T1}}\right)$ if small values are unfavorable. Correlations were determined by calculating the Pearson correlation coefficient. In order to test the efficacy of the training at each level in the patients, we compared the STs at the start and end of the training for each level of difficulty. For this purpose, dependent (paired) sample t tests were conducted. The transformed data were tested parametrically.

We used the Mann-Whitney U test for independent samples to compare the STs of the patients with the STs of the comparison group.

#### Eye movement analysis during on-screen visual search

The main model in the on-screen VS task was a mixed model repeated-measures (MMRM) ANOVA with STs as dependent variable, time (T1 and T2) as within-subject factor, and group (patients and healthy children of the comparison group) as between-subjects factor. Multiple linear regression analyses were performed at T1 for the STs with age and disease duration as factors.

Subsequent analysis was performed in the patients only to determine which eye movement variables caused the STs to become shorter. A repeated-measure ANOVA model with time (T1, T2 and T3) as independent variable was used for the following eye movement parameters:

- Amplitudes and durations of saccades (oculomotor parameters)
- Proportional number of saccades to the non-seeing side (eye movement strategy changes)

A Friedman test was used for the total number of saccades model.

#### Main model in the on-screen free viewing task

Viewing time was fixed in this paradigm, and the dependent variable was the proportional number of saccades to the non-seeing side, which was not available for the healthy subjects. Hence, performance was tested by a repeated-measures ANOVA, with time (T1, T2 and T3) as independent variable and proportional number of saccades to the non-seeing side as dependent variable.

Pairwise t statistics are reported for the significant effects as post hoc analysis, correction is not indicated. The Wilcoxon signed-rank test was used for the total number of saccades data.

#### Analysis of the table test data

A Friedman test was run to determine whether there were differences in STs in the table test for the patients between T1, T2 and T3. Pairwise comparisons in the patients were performed with the Wilcoxon signed-rank test. In order to test for differences in the comparison group, we used the Wilcoxon signed-rank test.

To compare table test STs of the patients with the STs of the comparison group, we used the Mann-Whitney U test.

#### All search tasks

For all search tasks (VST, on-screen and real life), we divided the patients into perinatal vs. acquired groups and the Mann-Whitney U test was used to indicate possible differences in STs at the different points in time: T1, T2 and T3.

#### Questionnaires

We ran Friedman tests in order to find differences in the scores of the patients for each domain of all questionnaires between T1, T2 and T3. We used the Mann-Whitney U test to compare the patients’ scores of the different domains with the scores of our comparison group.

For the KINDL questionnaire, a reference group (N = 2863) was already available from the BELLA study [36], so that we did not need to query our comparison group with this questionnaire. However, for the reference group of the BELLA study, only summary data (mean, standard deviation, sample size) were available for the different domains of the KINDL questionnaire. To compare the patients’ answers with the answers of the reference group of the BELLA study, we had to apply parametric methods to carry out this comparison and conducted independent-samples t tests using the summary data. Normality in the patients’ scores was tested with the Shapiro-Wilk test (p>0.05). If assumption of equal variances could not be met by the patients’ scores, Welch's unpaired t test was used [39,40]. We used software by GraphPad [41] to perform these calculations. It should also be noted that by using the values of the reference group of the BELLA study, the power of the test is much higher than by just using the results of our comparison group.

For the other questionnaires (IVI-C, CVAQC, specific mobility questions) we used the comparison group of normally sighted children in the present study. We transformed the raw CVAQC scores into logarithmic scores using a Rasch conversion calculator provided by the developers of the CVAQC tool [38]. Missing values in the IVI-C and CVAQC-25 questionnaires were replaced by the mean, as >75% of the scale values were available [42].

## Results

### Clinical parameters at baseline

The following symptoms were found at baseline (see Table 1):

- Head turn to the non-seeing side: 19 children (86%)
- Ipsilateral exotropia: 7 children (32%)
- Homonymous eccentric fixation: 6 children (27%)

These findings can be interpreted as spontaneous adaptations, where the head turn is considered a maladaptation. After training, a reduction of head turn was documented in 7/19 cases (37%).

### Search times during training

21 of 22 patients showed improved STs at least at one of the 3 levels of difficulty. The only patient who did not improve in any of the 3 levels (id 22), had Rasmussen’s encephalitis. This left him more impaired by his general status and cognition, and his STs scattered widely.

The individual STs and relative improvements at level 1 of the VST is shown in Table 3 for the patients and the comparison group. The patients’ relative improvement in STs was highest at level 1. Relative improvements for the patients at the three levels of difficulty were (mean relative improvement μri):

Level 1 –μri = 0.1721,

Level 2 –μri = 0.16,

Level 3 –μri = 0.1179.

**Table 3:** Individual search times and relative improvements at level 1.

	patients level 1			comparison group level 1			
id	ST_start_ (s)	ST_end_ (s)	ri	id	ST_T1_ (s)	ST_T2_ (s)	ri
1	12.9	6.6	0.49	100	2.7	2.8	-0.05
3	3.3	2.8	0.14	101	1.9	1.5	0.17
4	3.6	3.0	0.16	102	1.3	1.3	0.01
6	4.9	2.9	0.41	103	1.3	1.7	-0.23
7	3.7	2.5	0.34	104	1.5	1.5	0.03
8	8.6	7.7	0.10	105	1.5	1.6	-0.08
9	2.1	2.0	0.01	106	1.2	1.2	0.01
10	1.4	1.1	0.22	107	1.5	1.4	0.03
11	1.5	1.2	0.20	108	2.4	1.9	0.21
12	2.6	1.9	0.25	109	1.3	1.1	0.13
13	8.1	4.5	0.45	110	1.1	1.0	0.15
14	1.4	1.2	0.17	110	1.9	1.8	0.04
15	1.6	1.6	0.00	112	1.3	1.3	-0.01
16	2.9	2.2	0.25	113	1.1	1.0	0.08
17	3.4	2.4	0.29	114	3.3	3.0	0.10
19	1.8	2.0	-0.09	115	2.1	2.4	-0.14
20	1.5	1.2	0.18				
22	6.4	9.0	-0.41				
25							
28	2.6	2.2	0.14				
30	2.0	1.6	0.18				
31	8.6	6.8	0.22				

id = patient’s id, ST_start_ (s) and ST_end_ (s) = Search time per search target at start and end of training in seconds for the patients, ST_T1_ (s) and ST_T2_ (s) = Search times per search target in seconds for the comparison group at T1 and T2 without training, ri = relative improvement of search time ($\frac{{ST}_{start}-{ST}_{end}}{{ST}_{start}}$). Missing data: Patient #25 trained the entire 6 weeks only at level 2 (see Table in “S1 Table” for individual search times at levels 2 and 3).

More details about the individual data are shown in “S1 Table” in Supporting Information.

The mean relative improvement from T1 to T2 for the comparison group without training was μri = 0.0281. The mean relative improvement between T1 and T2 was 6.12 times greater for the patients than for the children in the comparison group without training.

The STs of the patients improved significantly from the start to the end of training at all three levels of difficulty. The dependent (paired) sample t test was found to be statistically significant:

Level 1 start of training (median = 2.88s, IQR = 1.71–5.67), level 1 end of training (median = 2.22s, IQR = 1.63–3.73)

Level 2 start of training (median = 4.41s, IQR = 2.89–8.91), level 2 end of training (median = 3.23s, IQR = 2.62–6.23)

Level 3 start of training (median = 3.04s, IQR = 1.92–3.84), level 3 end of training (median = 2.47s, IQR = 1.58–3.31)

Level 1: -0,081 (95% CI, -0.113 to -0.050) 1/s, t(20) = -5.42, p < 0,0001; Cohen’s d = 1.18

Level 2: -0.062 (95% CI, -0.095 to -0.029) 1/s, t(20) = -3.95, p < 0.001; Cohen’s d = 0.86

Level 3: -0.062 (95% CI, -0.089 to -0.036) 1/s, t(18) = -4.92, p < 0.001; Cohen’s d = 1.13

The effect sizes for these analyses were found to exceed the convention of Cohen’s d (1988) [43] for a large effect (d = 0.8).

The STs of the comparison group did not statistically differ between T1 (median = 1.48s, IQR = 1.30–2.04) and T2 (median = 1.52s, IQR = 1.19–1.85), -0,0254 (95% CI, -0,0660 to 0,0152) 1/s, t(15) = -1.33, p = 0.20; d = 0.33.

The Mann-Whitney U test indicated that STs at the end of the training at level 1 (ST = 2.22 s) were still significantly lower than the STs for the comparison group at T1 (Median = 1.48 s), U = 243.0, p = 0.021, z = 2.299 and T2 (median = 1.52 s), U = 252.0, p = 0.009, z = 2.575.

**Fig 4:**
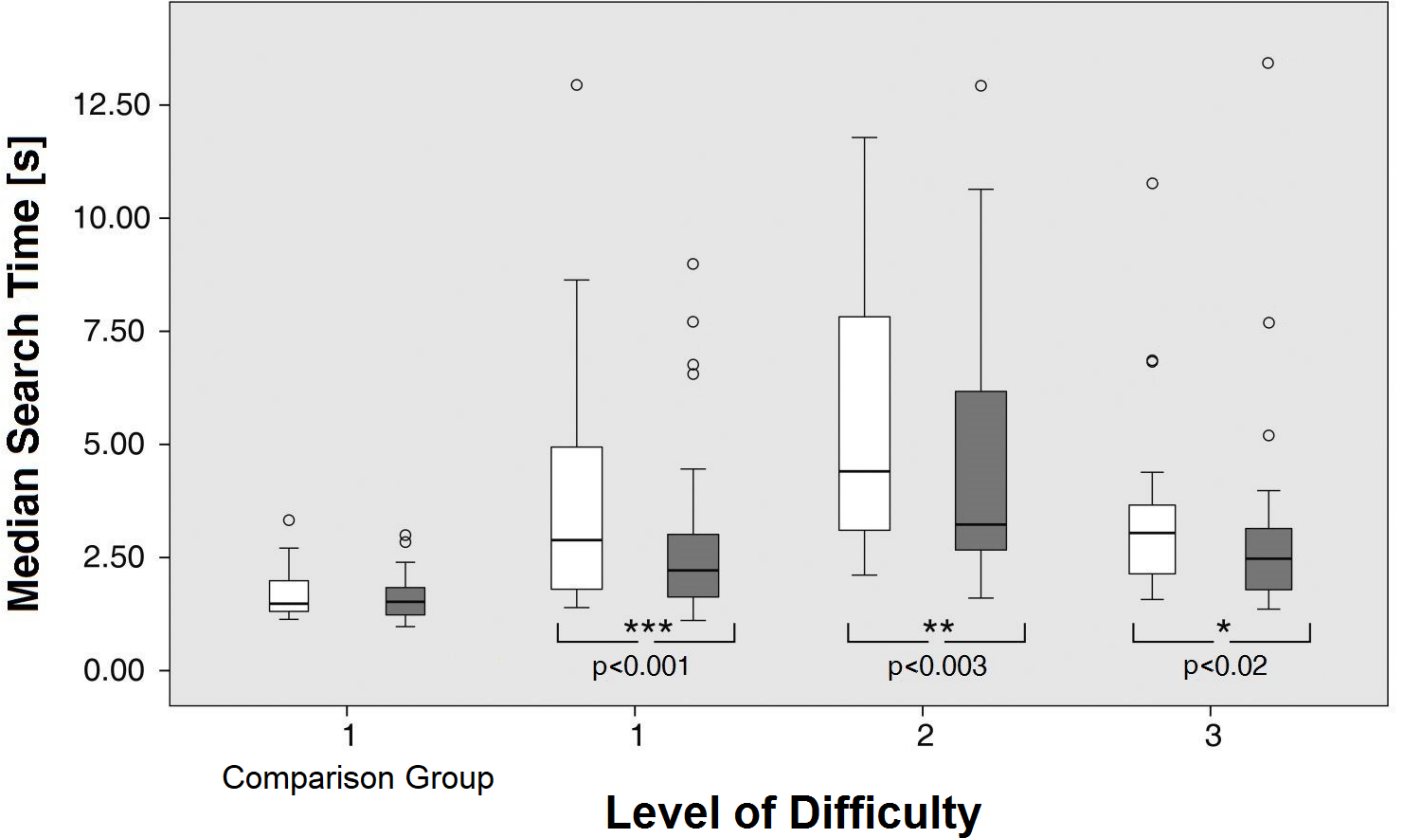
Search times during the training paradigm. In the healthy children of the comparison group, the STs were measured at T1 and T2 without training. The STs did not change by just repeating the task. The patients trained at three different levels of difficulty. The white boxplots show the STs at the beginning, the gray ones at the end of the training period of each level. The medians and IQRs indicate improvement in the patients at all three levels. Outliers were defined as values > 1.5 IQR above the 75% IQR.

Some patients reached normal STs at the end of the 6 weeks training. Fig 4 shows the results of the STs over the training period for all subjects, the main improvement occurred during the first 4 weeks. No such improvement was seen in healthy children of the comparison group, who performed one test at level 1 for 15 minutes at T1 and T2 without training in between. The IQR of the comparison group's STs is similar at T1 and T2 (0.74s vs. 0.66s, respectively), because the STs are quite close together. In contrast, the IQR of the STs of the patients at the beginning of the training is rather large (IQR = 3.95s). There are children with HH who start with similar STs as the comparison group, and there are children whose STs are far away from those of the comparison group. After the training at level 1, their IQR is reduced considerably (IQR = 2.11s). Yet the patients, on average, did not reach the STs of the comparison group. STs at baseline correlated negatively with age at examination (R = - 0.724) and positively with improvement during training at level 1 (R = + 0.702).

**Fig 5:**
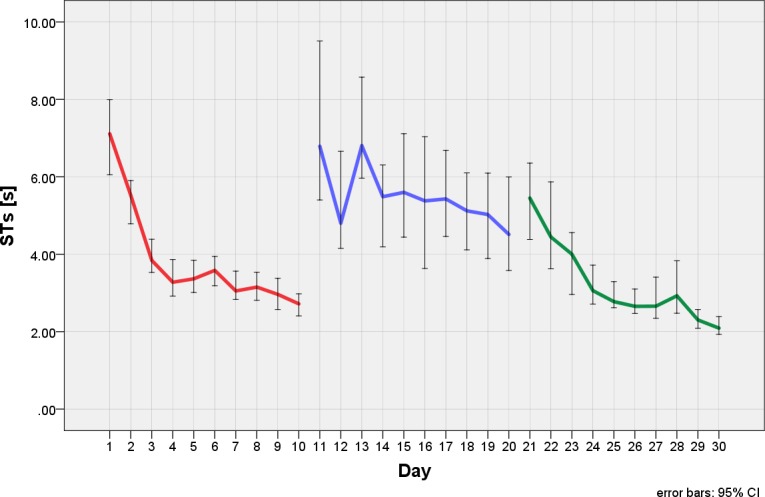
Example of a representative child with HH during training at three different levels of difficulty (red, blue and green curves) showing improvement of search times at all levels. The asymptotic curves at each level show that improvements from the training cannot be increased by further practice.

Fig 5 depicts a representative example showing an asymptotic curve indicating that further training is unlikely to improve STs.

### On-screen visual search: Eye movements

**Fig 6:**
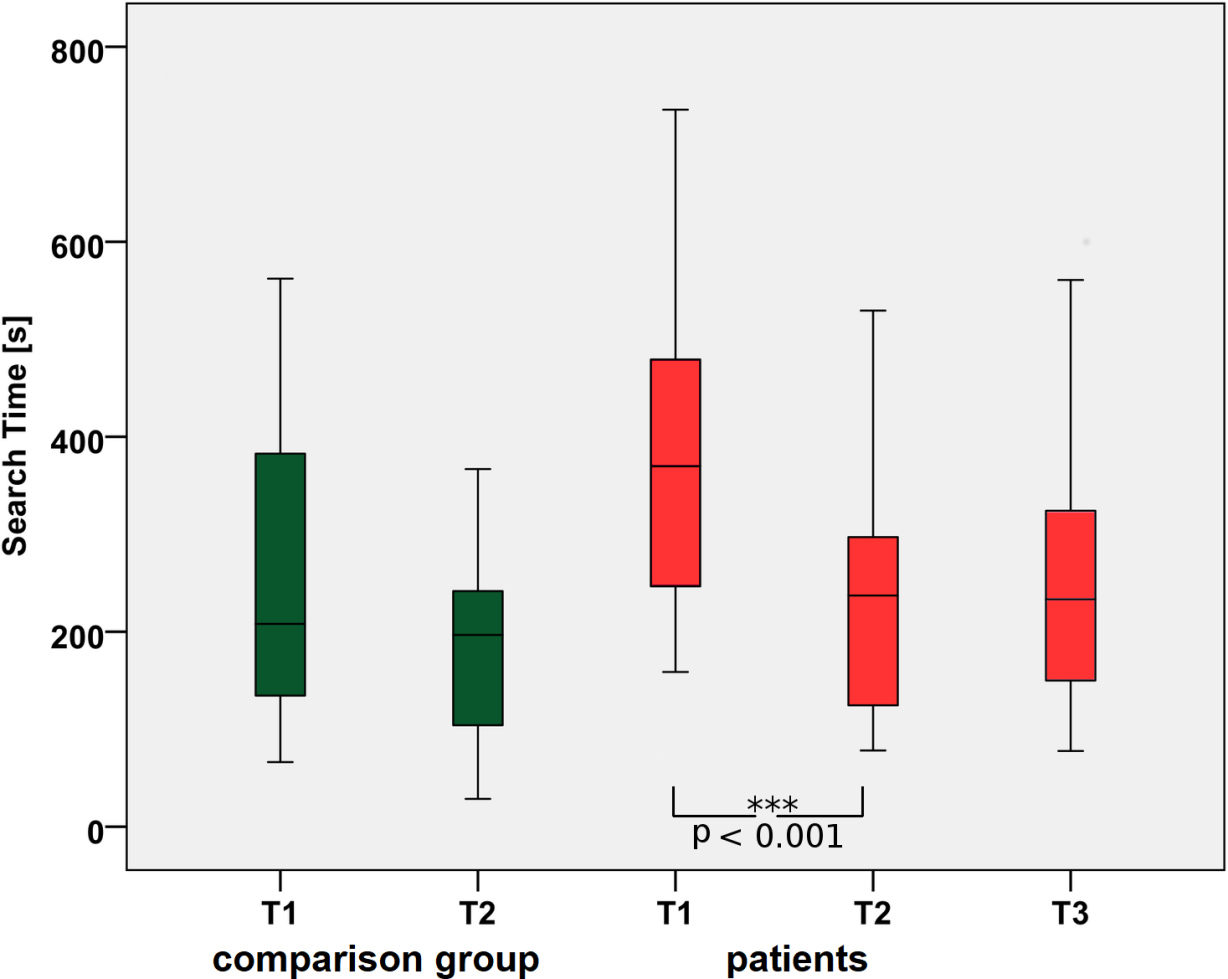
Search performance during eye tracking. Improvement of STs from T1 to T2: The effects are maintained at T3, which supports the hypothesis that the newly learned strategies persist because they keep being used in everyday life.

The main variable in the VS task was the sum of all STs in seconds/session. Fig 6 shows that the patients improved significantly after training. Their mean summed STs were 373.6 +/- 39 (SEM) s at T1 vs. 242.7 +/- 28 (SEM) s at T2. For the comparison group (without training), these values were 237.4 +/- 36 s at T1 vs. 187 +/- 27 (SEM) s at T2, a considerable difference that was statistically not significant (p = 0.2). Here, time and group were both significant factors (F = 16.7, p<0.001 and F = 5.084, p = 0.03, respectively), while the interaction “time” x “group” was not significant (F = 6.99, p = 0.09).

In the patients, the level of performance after training (242.7 +/- 28 SEMs) at T2 was sustained six weeks after the end of the training (244.5 +/- 32 SEMs) at T3 (p < 0.01 for the T1 vs. T2 and p = 0.06 for the T2 vs. T3 comparisons). The patients' performance was close to that of the comparison group at T2 (Fig 6). The improvement in patients after training is also reflected in the decreasing number of targets that were not found during the maximum ST of 60 s (T1 = 13%; T2 = 5%; T3 = 4%).

For the patients, testing the oculomotor parameters showed that time was not a significant factor for the average saccade durations (F(2,34) = 1.3, p = 0.3). This indicates that there was no significant difference between average saccade durations at T1 (M = 26.8 s, SEM = 2), T2 (M = 31.9 s, SEM = 2) and T3 (M = 28.8 s, SEM = 2). However, there was a significant effect of time on the average saccade amplitudes (F(2,34) = 3.7, p = 0.04). The pairwise t tests showed that there were significant differences (p = 0.04) between the average saccade amplitudes at T1 (M = 6.6, SEM = 0.6) and T2 (M = 7.9, SEM = 0.5), which persisted at T3 (M = 7.6, SEM = 0.5). A significant effect (Friedman’s χ^2^(2) = 12.1, p = 0. 002) was also found for the total number of saccades in the patients at the different points in time. Post hoc analysis revealed a significant decrease in number of saccades from T1 (median = 1778, IQR = 1324–2621) to T2 (median = 1172, IQR = 1631–695): p<0.01. The difference persisted at T3 (median = 1133, IQR = 1547–747).

**Fig 7:**
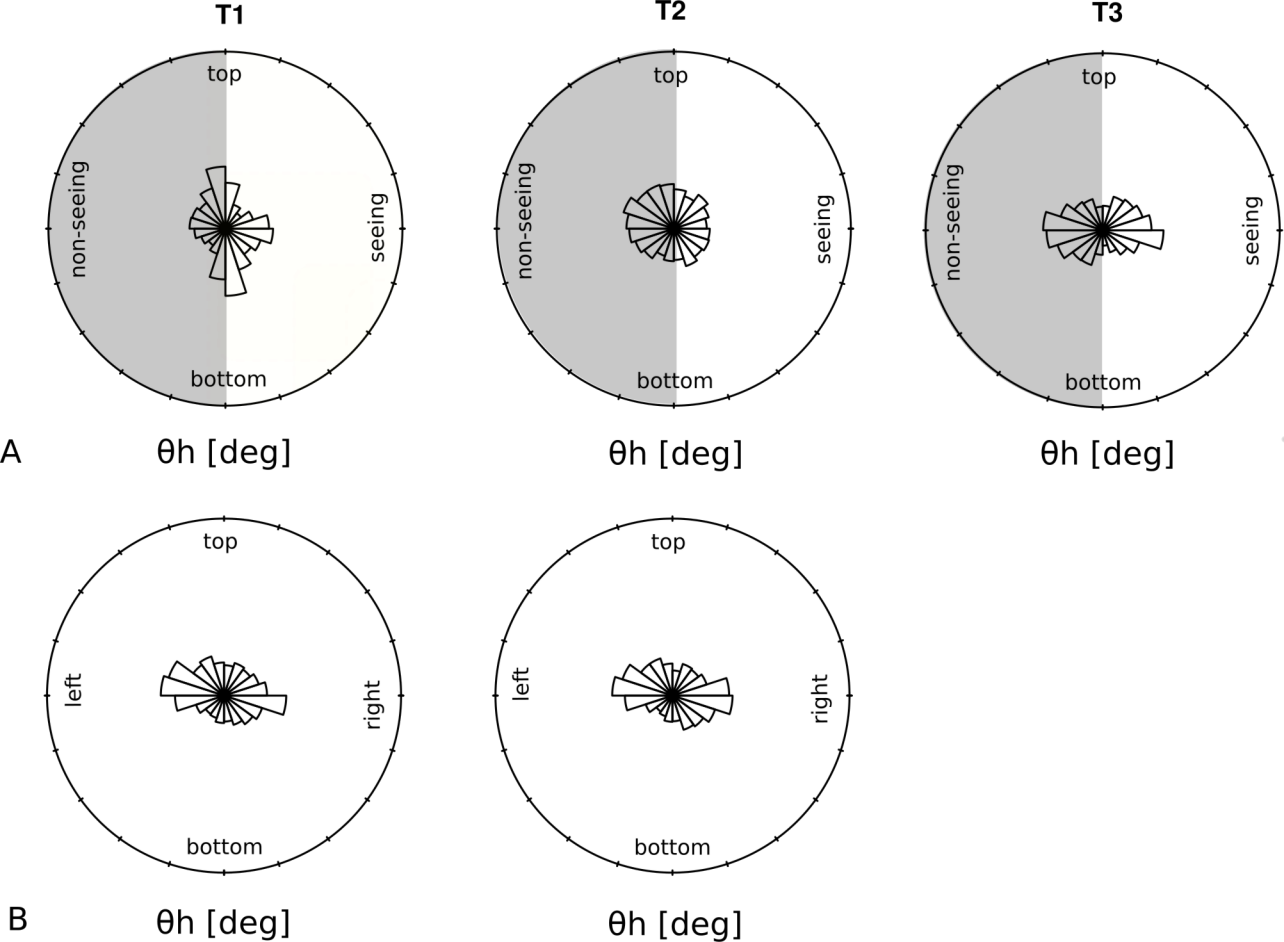
Directional characteristics of the eye movement pattern. The distribution of the wedges shows the angles theta (θ_h_) between the saccades and the horizontal meridian. A: Changes in eye movement patterns during VS for one patient with major improvement in STs (only 6 months after onset of HH). Before training, the patient showed many vertical saccades, while horizontal saccades were mostly directed towards the seeing hemifield (white). At T2, the dominance of vertical saccades has disappeared, and more horizontal saccades are directed towards the non-seeing hemifield (gray). At T3, a prevalence of horizontal saccades has developed, so that the search pattern now resembles the pattern of the healthy subjects. B: Search patterns of one healthy subject at T1 and T2. The length of each radial wedge indicates the number of saccades in that direction without change just by repeating the task.

Our analysis of the proportional number of saccades to the non-seeing side showed a significant effect of time (F(2,34) = 3.9, p = 0.03) indicating a significant (p = 0.02) difference between T1 (M = 0.51, SEM = 0.02) and T2 (M = 0.56, SEM = 0.02). This is graphically shown in Fig 7A, which plots the eye movement pattern of the patient who improved the most after the training. For the different points in time (T1, T2 and T3), the figure plots the directional characteristics of the eye movement pattern represented by the distribution of the horizontal angle (θ_h_) that corresponds to the angle between a saccade and the horizontal meridian. It is apparent that before training (T1), the angle distributions exhibit spatial anisotropy along the vertical meridian, and the patient performed more saccades towards his seeing hemifield (right). The anisotropy is not present at T2, where the eye movement distribution shows a uniform pattern. Six weeks after training (T3), the pattern resembles the scanning strategy of a typical healthy observer, shown in Fig 7B. It is also apparent that the proportion of horizontal saccades performed by the patient to the non-seeing hemifield (left) is higher after the training at T2 and remained at the same level at T3 (M = 0.53, SEM = 0.02).

Older children showed shorter STs (F(3,14) = 11.9, p<0.001, with adjusted R^2^ of 0.7), while disease duration was not a significant factor. There was no correlation between age and STs in the healthy children of the comparison group (R = -0.113, p = 0.677).

During FV, we analyzed the proportional number of saccades to the non-seeing side. It was found that the relative number of saccades to the non-seeing side did not differ at T1, T2 and T3 for the different subjects (F(2,34) = 0.238, p = 0.8).

**Fig 8:**
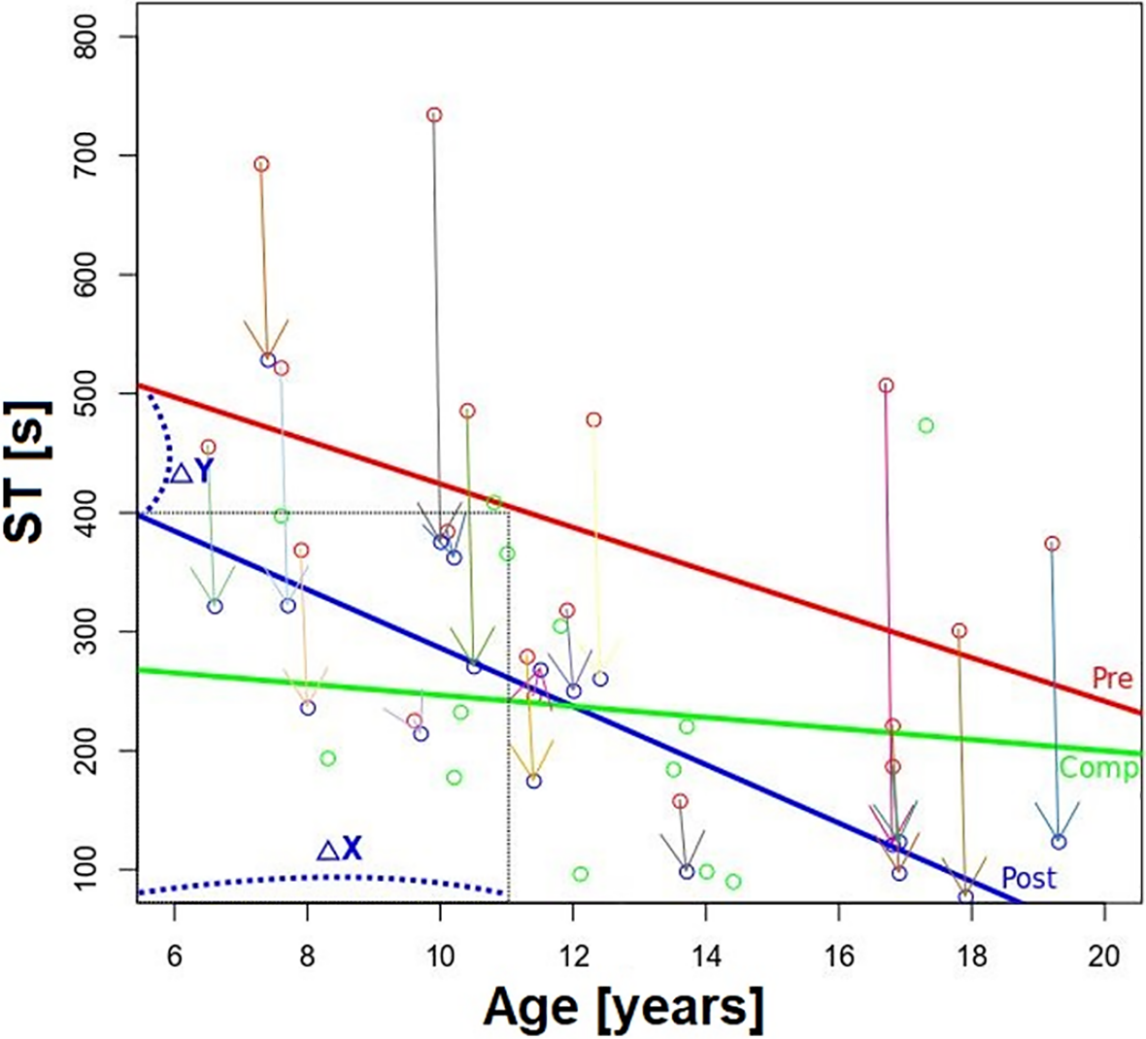
The effect of training vs. spontaneous adaptation and natural development by age. Red and blue circles represent performance before (Pre) and after (Post) training, while color-coded arrows indicate individual ST gains after training. The red line is the linear fit between age and ST before training and represents the spontaneous adaptation and natural development in children with HH. The blue line is the linear fit between age and STs **after** training. The amount of ST improvement obtained by training (delta Y, blue stippled line) is about 130 s (average over all patients) and could theoretically only be achieved after approximately 6 years of spontaneous adaptation (delta X). The green line represents the STs of the healthy children, showing only a small decrease with natural development. Arrows represent individual improvements of the patients before and after training.

We wanted to know to what extent the ST improvement during on-screen testing with eye tracking in the patients could be explained by training, or by spontaneous adaptation and natural development. It was found that the amount of ST improvement obtained after training is about 130 s, which a patient could theoretically only achieve by approximately 6 years of spontaneous adaptation and natural development (see Fig 8).

#### Summary of the eye movement results

On-Screen VS Task:

Significant changes:

- STs improve from T1 to T2, remain stable at T3
- saccade amplitudes increase from T1 to T2, remain stable at T3
- total number of saccades decreases from T1 to T2, remains stable at T3
- fewer not-found targets
- proportional number of saccades to the non-seeing side increase from T1 to T2, but go back to the initial level at T3

No changes:

- average saccade duration

On-Screen Free Viewing Task:

- proportional number of saccades to the non-seeing side: no differences at T1, T2, T3

### Daily living

#### The table test

**Fig 9:**
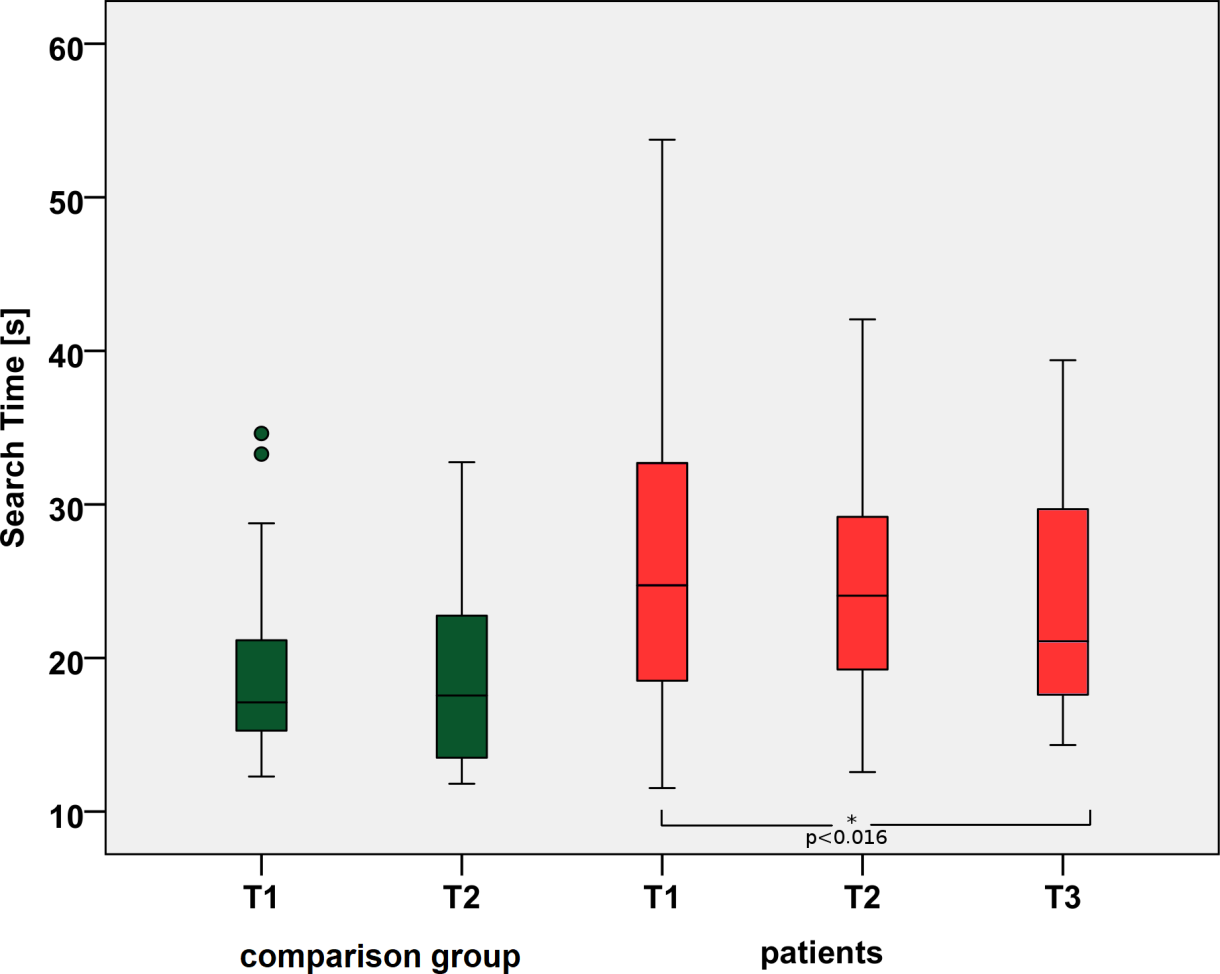
Search times during a real life search task in the table test. The median STs for the patients improve significantly from T1 to T3 with p<0.016. T1 = baseline and before training in patients, T2 = after training in patients, T3 = follow-up after 6 weeks.

The STs for patients in the table test (see Fig 9) were significantly different between the three times (Friedman’s χ2(2) = 6.095, p < 0. 047). Post hoc analysis revealed a significant difference in STs from T1 (median = 24.72 s) to T3 (median = 21.09) (p = 0.016).

For the comparison group, we found no statistically significant difference between T1 (median = 17.11) and T2 (17.56 s) (Wilcoxon signed-rank test, z = -0.84, p = 0.401).

The STs for the patients at T1 (median = 24.72 s) were significantly longer than the STs for the comparison group at T1 (median = 17.11, Mann-Whitney U test, U = 250.5, p = 0.028, z = 2.203).

At T2, there was no significant difference between the patients (median = 24.06 s) and the comparison group (median = 17.56 s, Mann-Whitney U test, U = 127, p = 0.067).

The patients’ STs at T3 (median = 21.09, Mann-Whitney U test) yielded no significant difference relative to those of the comparison group at T1 and T2 (U = 231.5, p = 0.052, and U = 113, p = 0.157, respectively).

#### For all search tasks

We did not find significant differences between the STs in children with perinatal vs. later acquired HH for any of the search tasks: **VST** (U = 34.0, T = -0.495, p = 0.7), **on-screen** (U = 38.0, T = -0.165, p = 0.9), and **table test** (U = 44.0, T = 0.118, p = 0.9).

#### Questionnaires

The analysis of the questionnaire data showed, in principle, similar results. Within the patient group, there was always an improvement between the different times (except in the domain “School”). Although this result did not reach statistical significance, the change of the scores developed always in the same direction. For the domain “Friends” (KINDL) and the domain “Social” (CVAQC), the improvement within the patient group was significant.

In comparison to the reference group of the BELLA study (KINDL) or our comparison group (all other questionnaires), we observed a statistically significant impairment at T1 and T2 in the domain “Friends”, “Emotional wellbeing” (KINDL) and “Total Quality of Life” (IVI-C), which disappeared at T3. In the domain “Self-esteem” (KINDL, children < 13 years), the patients started even better than the reference group, without statistically significant difference at T1, but the difference became statistically significant at T2 and T3. In the domain “Mobility” (CVAQC), the initially significant difference relative to the comparison group did not exist any more at T2 and T3.

The following section shows the analysis of the questionnaires in detail:

KINDL, parents’ answers:

For the KINDL questionnaire, there are different forms according to the age of the children, which is why we divided our cohort of children into those equal or younger than 13 and those older than 13 years of age (see Methods).

No statistically different changes were observed (Friedman-test, p > 0.05) between T1, T2 and T3 for the parents’ answers to the KINDL questionnaire (all 7 domains).

Domain “Family”:

The parents’ answers in the domain “Family” increase from T1, T2 to T3, but do not reach statistically significant improvements. Yet, at T3 (M = 83, SD = 8.6, n = 22) only, the parents rated the score for the domain “Family” statistically significantly higher than the reference group of the BELLA study (M = 77.7, SD = 14.3), t(21) = 2.8604, p = 0.0094.

Domain “Friends”:

The parents rated the interaction of their children with friends significantly lower at T1, T2 and T3 than the parents of the reference group in the BELLA study (M = 78.0, SD = 13.4, n = 2863):

T1: (M = 63.4, SD = 14.7, n = 22), t(21) = 4.6437, p < 0.0001.

T2: (M = 62.8, SD = 16.4, n = 22), t(2883) = 5.2906, p < 0.0001.

T3: (M = 63.7, SD = 13.9, n = 22), t(2883) = 4.9849, p < 0.0001.

KINDL, children’s answers:

Domain “Friends”, children of 13 years or younger:

The domain “Friends” of the patients was found to be statistically significantly different between the three times (Friedman’s χ2 (2) = 7.518, p < 0. 020). Post hoc analysis revealed a statistically significant improvement from T1 (median = 63.6) to T3 (median = 74.2) (p = 0.022).

Whereas the scores in the “Friends” domain were significantly higher for the reference group of the BELLA study (M = 80.9, SD = 13,4, n = 747) at T1 and T2, but no longer at T3:

T1: (M = 63.6, SD = 17.6, n = 22), t(760) = 4.9180, p = 0.0001.

T2: (M = 71.0, SD = 19.4, n = 22), t(760) = 2.8049, p = 0.0052.

T3: (M = 74.2, SD = 15.5, n = 22), t(760) = 1.9114, p = 0.0563.

The same trend was observed in the children’s group older than 13:

Domain “Friends”, children of over 13 years:

In this group of older children, the improvement in the domain “Friends” did not change significantly between the different times (Friedman test, p>0.05).

Compared to the reference group of the BELLA study (M = 75.2, SD = 15.0, n = 1148), the score for the domain “Friends” was not statistically different any more at T3, while at T1 and T2 it was:

T1: (M = 50.9, SD = 20.5, n = 7), t(1153) = 4.2635, p = 0.0001.

T2: (M = 63.4, SD = 31.5, n = 7), t(1153) = 2.0568, p = 0.0399.

T3: (M = 64.6, SD = 12.3, n = 7), t(1153) = 1.8656, p = 0.0624.

Domain “Self-esteem”:

Scores for the domain “Self-esteem” improved from T1 to T2 and T3, but did not reach a statistically significant level. The mean scores were higher in the patients at T1, T2 and T3 compared to the reference group of the BELLA study. The scores did not differ significantly from the reference group at T1. At T2 and T3, the score increased further and reached statistically significantly higher values compared to the reference group in the BELLA study (M = 56.3, SD = 18.5, n = 747), i.e. the patients had higher self-esteem than the reference children:

T1: (M = 64.7, SD = 18.5, n = 15), t(760) = 1.7255, p = 0.0848.

T2: (M = 67.9, SD = 17.7, n = 15), t(760) = 2.4063, p = 0.0164.

T3: (M = 76.3, SD = 19.5, n = 15), t(760) = 4.1414, p = 0.0001.

Domain “Emotional wellbeing”:

The score in the domain “Emotional wellbeing” remained stable from T1 to T2, then increased further, and finally reached the scores of the reference group at T3. While at T1 and T2 the difference to the reference group (M = 83.2, SD = 10.7, n = 747) was statistically significant, it was not different any more at T3:

T1: (M = 75.0, SD = 16,2, n = 15), t(760) = 2.9044, p = 0.0038.

T2: (M = 74.6, SD = 17.3, n = 15), t(760) = 3.0373, p = 0.0025.

T3: (M = 79.2, SD = 18.2, n = 15), t(760) = 1.4092, p = 0.1592.

Domain “School”:

At T1, the rating of the children’s group equal or younger than 13 years were significantly better than the values of children from the reference group (t(760) = 2.4769, p = 0.0135). The score decreased and was not significantly different from the reference group any more at T2 and T3 (t test, p>0.05).

Impact of Vision Impairment for Children (IVI-C):

There were no significant changes for the patient group in the five domains (mobility, interaction with friends, school, emotional wellbeing, total quality of life), Friedman-test, p>0.05.

The domains “Mobility” and “Emotions” improved slightly, but the changes did not reach statistical significance.

For total quality of life, the achieved score was significantly lower in the patients than in the comparison group (median = 94) at T1 (median_T1_ = 72, U = 72, p = 0.037), and T2 (median_T2_ = 78, U = 78, p = 0.030). At T3, a Mann-Whitney U test showed no significant difference any more between the patients (median = 81) and the comparison group (median = 94, U = 70, p = 0.056).

The 25-item Cardiff Visual Ability Questionnaire for Children (CVAQC- 25):

The resulting scores in the patients ranged from –3.15 (higher visual ability) to +0.40 (lower visual ability). The domain “Social” of the patients was found to be significantly different between the three times (Friedman’s χ2(2) = 6.937, p = 0.031). Post hoc analysis revealed a significant difference from T1 (median = -1.91, IQR = -2.26 to -0.15) to T2 (median = -1.91, IQR = -2.26 to -1.27) (p = 0.018). For the domain “Mobility”, the achieved score was significantly worse in the patients (median = -0.65, IQR = -2.65 to 0.9) than for the comparison group (median = -2.48, IQR = -3.24 to -2.09) at T1, Man-Whitney U test, U = 27, p = 0.032. After training at T2 and at T3, Mann-Whitney U tests showed that there were no significant differences any more between the patients (median_T2_ = -1.59, IQR = -2.76 to -0.13; median_T3_ = -1.67, IQR = -2.67 to -0.28) and the comparison group (median = -2.48, IQR = -3.24 to -2.09), at T2 (U = 35, p = 0.115) and T3 (U = 32, p = 0.110).

Domain “School”:

For the patient group, there were no significant changes (Friedman test, p>0.05).

The Mann-Whitney U test showed significant differences between the patients and the comparison group (median = -2.90, IQR = -3.05 to -2.06) at T1 and T3, but not at T2.

T1: (median = -1.10, IQR = -2.77 to 0.40), (U = 27.5, p = 0.034).

T2: (median = -2.77, IQR = -2.92 to -0.79) (U = 44.0, p = 0.325).

T3: (median = -1,10, IQR = -2.38 to -0.79) (U = 22.0, p = 0.030).

Specific mobility questions (see Table 4):

At T1, the parents reported more problems of their child getting out of the way of something or somebody than the children themselves (88% vs. 67%). At T3, children and parents reported fewer problems (59% of the parents and 57% of the children). Difficulties with bumping into objects were reported by 65% of the parents and 56% of the children at T1 and 41% of the parents and 44% of the children at T3. In both conditions, there was further improvement between T2 and T3.

**Table 4:** Specific mobility questions to children and parents.

Problems:		Children n = 18	Parents n = 17
Getting out of the way	T1	66.7	88.2
	T2	66.7	76.5
	T3	55.6	58.8
Bumping into something	T1	55.6	64.7
	T2	50.0	52.9
	T3	44.4	41.2

Percentage of positive answers

Independent of the questionnaires, some individual patients reported regaining abilities and fun in playing tennis or bowling, and in feeling more comfortable in complex surroundings at school (schoolyard and stairwell).

## Discussion

### Clinical parameters at baseline

At baseline (T1), we found head turn to the non-seeing side in 19 children (86%) with improvement in 7 cases (37%), while Koenraads et al. [23] reported a lower prevalence (53%). Head turn can be the only visible sign of HH in children, because the field defect often remains undetected or cannot be properly assessed due to insufficient cooperation. The prevalence of unrecognized visual field defects in children with brain tumors has been reported to be surprisingly high (15%) [44]. Head turn alone is a maladaptation, because it can lead to torticollis and does not enlarge the functional visual field without scanning eye movements [25].

Seven children showed ipsilateral exotropia (31.8%). A similar prevalence (38%) was reported by Koenraads et al. [23] after hemispherectomy. Ipsilateral exotropia was also described in several case reports [26,27,45]. It is important to be aware of the field defect, because in cases with anomalous correspondence, strabismus surgery is contraindicated [27,45]. Homonymous eccentric fixation occurred in 6 children (27.3%). We detected this behavior in about 20% of adults with HH before [46,47]. This shift of fixation by 1–1.5° is a valuable adaptation especially for reading, because it shifts the field defect towards the non-seeing side and creates an additional narrow seeing strip along the vertical midline.

### Training parameters

The majority of the patients (21/22) showed improved STs during training. In the patients, the highly significant improvement of the STs at all levels of difficulty took place mainly in the first 4 weeks of the training (Level of difficulty 1 and 2). At level 3, the children already started with lower STs and showed less improvement (see Fig 4), which may be a saturation effect of the training. Hence, four weeks of training would already have been sufficient to achieve the shortening of STs.

The finding that STs at baseline correlated negatively with age at examination indicates increasing spontaneous adaptation as well as natural development with advancing age. The fact that STs at baseline correlated positively with improvement during Training at difficulty level 1 shows that less adapted children have a higher potential for improvement in contrast to well-adapted children, who started with normal or near-normal STs. The observation that the median STs on the seeing side of patients differ also from the healthy children of the comparison group at baseline could be explained by generally slower search caused by their neurological condition.

### Eye movements during on-screen visual search

Previous reports by others, as well as our own study on adults with HH, indicate that we could expect an effect of the training on some eye movement parameters [2,12,48–51]. This could prompt questions about the role eye movements play in performance improvements through training. On the one hand, there may be changes in the basic oculomotor apparatus caused by practice. Furthermore, it is conceivable that higher order mechanisms may be involved, e.g. a new gaze strategy, or perceptual learning.

Our results showed that, STs significantly improved after training, which was sustained also 6 weeks after end of the training (Fig 6). The shorter STs after training can be accounted for by the fact that significantly fewer saccades had to be performed to solve the task. This indicates that the training taught the patients an efficient strategy for directing their gaze, as average saccade amplitudes increased from T1 to T2 and persisted at T3. The fact that saccade durations did not change indicates that no changes in the basic oculomotor apparatus occurred.

Along with the changes of the total number of saccades and their amplitudes, we also found that patients, on average, performed more saccades to the non-seeing side after training (at T2). These findings indicate that the change in search performance after training could be caused by improved attentional and scanning abilities [52,53]. The finding that patients performed more saccades to the non-seeing side is in accord with a previous study that suggested that the strategy to perform more saccades into the non-seeing field is advantageous for the patients [54] and could also yield a functional benefit through shorter STs. Altogether, the patients’ search performance became more effective by performing fewer and larger saccades, as well as proportionally more saccades to the non-seeing side.

It has been suggested that an initial larger (hypermetric) saccade brings more of the lost field into view and that this scanning strategy is effective to partially compensate for HH [46,55]. Our finding that the average saccade amplitudes increased after training, which was sustained at follow-up, shows that the saccades towards the non-seeing side brought more of the lost hemifield into view.

The decrease of the number of saccades during the search task after training is in agreement with Mannan et al. [48], who found that adult patients with HH also required fewer saccades to locate the target after training VS. This can be interpreted as a more effective search strategy, which is corroborated by the finding that our patients, on average, found more targets on the screen after training (+8%).

We found no evidence for better spontaneous adaptation of the visual system in children with very early (perinatal) vs. later acquired brain lesions. This is in contrast to the common assumption based on anecdotal reports and a previous study [29].

At baseline, STs for the congenital and acquired brain lesions did not differ significantly. However, we found that with advancing age the children perform better, regardless of the time of injury. Since advancing age and the age at brain injury are interrelated, the age at examination of the children with perinatal lesions is a confounding factor that was not considered in the study by Tinelli et al. [29] and may also have influenced the better performance in the children with perinatal lesions.

During free viewing (FV), the children with HH did not perform more saccades into their non-seeing hemifield at baseline, and the total number of saccades of patients and healthy children did not differ significantly, neither before nor after training, even though there were large inter-individual differences. As stated above, the basic eye movement parameters were not different between the two groups at baseline. Both these facts together can be interpreted as an adequate spontaneous adaptation by children with HH who developed strategies similar to those seen in healthy children for scanning the visual world in a FV task, so that the eye movement parameters were balanced between both sides.

Furthermore, we analyzed whether the effect of VST demonstrated before in a randomized controlled trial in adult hemianopes, may be extended towards children. First, from the asymptotic curves in Fig 5, it is evident that at each level of task difficulty, children’s performance reached a plateau before the end of the training, so that further practice of the training task would have been unlikely to further shorten the STs. This finding directly demonstrates that the VST was efficient regarding the task to find the target among the distracters. Second, if reductions in STs in the training task are compared between adults [2] and children with HH, it is evident that both groups improved significantly and the reduction in the adults is 47% (pre/post training), while, under conditions similar to the previous study, it is about 28% in children. However, it is worth noting that, on average, children started at significantly shorter STs before training and therefore had a narrower margin for improvement. Taken together, these findings provide critical quantitative information about the amount of ST reduction after VST for the hemianopic children and suggest that the training was successful.

A crucial question is whether the improved performance in the training transferred to the realm of daily living and benefited the patients in those activities. The training employed here, as well as in the study by Roth et al. [2], was not “supervised”, as we did not instruct the patients how to move their eyes. Instead, we relied on the children’s potential to spontaneously acquire a more successful strategy [56] to find the targets in the non-seeing hemifield. Since we could not control what they did during the training, it was possible that a patient started with one strategy that was later developed further.

Therefore, improved performance in the patients can be due to the increased saccade amplitude combined with decreasing absolute number of saccades.

The increased proportional number of saccades to the non-seeing side at T2 may additionally contribute to more efficient scanning, but influences search performance less than saccade amplitude.

The lack of a persistent increase of the proportional number of saccades to the non-seeing side at follow-up suggests that the VST effect for this variable is temporary, at least for some children. Therefore, for those children, it could be helpful to continue with the VST after T2 to maintain the learned strategies. In addition, the duration of the effect is different in different tasks, where the role of attention may also vary. The on-screen search task and the table test are different and may require different attentional demands, which might account for the differences in search performance at T2 and T3.

Our study on adults [2] has shown that the *average* effect of the training can be transferred to activities of daily living, but not all adults are the same and may develop different strategies. Children have been shown to possess equal or, in some tasks, higher capacity of working memory, brain plasticity, and ability to learn than adults [56,57]. Therefore, they may actually be more likely to benefit from the training. This suggestion is supported by the finding that the young patients’ performance after training improved in the VS task. In addition, their search times after training did not differ significantly any more from those of the healthy children, whose STs did not change just by repeating the task once. In addition, the results of our table test represent a typical daily task and showed significant long-term improvement after training in the children with HH.

One could also argue that the VST may not have been the main factor responsible for the improvement in performance, which could rather be due to spontaneous adaptation or natural development during the VST. To rule out this possibility, we compared the amount of ST improvement in the VS task due to spontaneous adaptation and natural development (STs before training) with the amount of ST improvement obtained after VST (see the blue line in Fig 8). ST (the y-axis in the figure) is plotted as a function of age at examination (x-axis). From the equation for the linear fit (y = 606–18 x) we can conclude that in the range of ΔY = 130 s (the average over all patients), it would take approximately 6 years (delta X) of spontaneous adaptation and natural development in order to achieve the same ST improvement. Since VST lasted no longer than 6 weeks in our paradigm, we can conclude that the improvement was due to the training, rather than to spontaneous adaptation.

The time since injury could also be a parameter for assessing spontaneous adaptation, where we would have expected a correlation. The fact that we did not find a correlation could be due to the confounding factors of age at disease onset, disease duration, and the age at examination. Since we found that the latter is a significant predictor of performance, the age at disease onset could also play an important role in the amount of spontaneous adaptation and natural development assessed at T1. However, we were unable to test the patients’ performance at the time of disease onset, so that we cannot disentangle the amount of contribution from disease duration and age at disease onset as components contributing into the final improvement. However, as we show in Fig 8, the *improvement* line (in green) of STs of the healthy children is more or less flat and lacks a clear correlation with age. On the other hand, the corresponding line for the patients before training showed some slow improvement in the sense of spontaneous adaptation and natural development, but the line for the patients during and after training showed a marked decrease of STs indicating a training effect. Therefore, we believe that the improvement after the training period is caused by the training and not only by natural development by age or spontaneous adaptation. From the individual data in Fig 8 it is evident that although almost all patients improved, the ones who started with shorter STs at T1 improved less at T2. This means that the improvement of STs by the faster patients may have contributed less to the observed positive correlation between baseline STs (red data fit) and improvements in STs (blue data fit) after VST. Note that the ST at T1 is a strong predictor of improvements after VST at T2 but we could not control for this confounding variable without a control group.

### Daily living

#### Table test

In the table test, the improvement of STs in the patient group was not significant at T2, but was significant at T3. However, the comparison with the healthy children shows that the patients improve also between T2 and T3: the difference to the comparison group is significant at T1, but no longer at T2 and T3. Note that this test is semi-quantitative and the precision of measurement depends on the experience of the examiner.

#### Quality of life questionnaires

Within the group of patients, the scores revealed statistically significant improvements in two domains:

Interestingly, the domain “Friends” improved for the children aged 13 years or younger (KINDL) and the domain “Social” from CVAQC. Yet, we have to point out the problem of multiple testing when comparing domains.

In addition, some individual reports regarding sports, safety, and social life were positive. Regarding the other children, it was our impression that some did not understand the questions, so that they could not relate them to their vision problem.

Compared with the healthy children of the reference group of the BELLA study and our comparison group, several scores showed statistically significant differences at T1, which disappeared at T2 and T3. Especially mobility improved until T3 to normal values in the CVAQC questionnaire, which corresponds well to the reduced mobility problems in our special mobility questions.

## Conclusions

In summary, we could show an effect of the training regarding several outcome measures, especially our primary outcome, ST.

The majority of the patients in this study (21/22) showed an improvement of search performance during training. Spontaneous adaptation of a gaze strategy in children may be good enough for free viewing but not optimal for VS. The benefit for the children is indicated by significant improvement of various variables: Decreasing STs during all search tasks, larger saccade amplitudes, decreased number of saccades, and increased proportional number of saccades to the non-seeing side. We have shown that capabilities developed during computer-aided training can be applied to everyday tasks, which led to shorter STs in the table test. These findings were augmented by individual subjective reports of improvements in daily living. We suggest that improved performance in the VS task was based on learning better strategies. We conclude that training of VS tasks for children with HH should be considered as a mode of therapy since spontaneous adaptation alone is often not sufficient to help with activities of daily living.

Future studies employing a control group of patients will have to be conducted to solidify the demonstrated benefits of VST for children with HH. Further research should also focus on identifying additional outcome measures for activities of daily living and QoL that more directly assess the specific vision-related needs of children with HH.

## Supporting information

S1 Table Individual search times (at the start and end of each level of difficulty) and relative improvements in patients and comparison group at T1 and T2 without training. (DOCX)

S1 File Trial study protocol in original German language. (DOC)

S2 File Trial study protocol in translated English language. (DOC)

S3 File The TREND statement checklist. (PDF)
