# Supplementary material for: Effects of visual search training in children with hemianopia
Source: PLoS One. 2018 Jul 18;13(7):e0197285. doi: 10.1371/journal.pone.0197285 (PMC6051578; doi:10.1371/journal.pone.0197285)
Supplement: S1 Table — (DOCX) [file pone.0197285.s001.docx]

**S1 Table. Individual search times (at start and end of each level) and relative improvements in patients and comparison group at T1 and T2 without training.**

|  | Level 1 | | | Level 2 | | | Level 3 | | |
| --- | --- | --- | --- | --- | --- | --- | --- | --- | --- |
| id | ST_start_ | ST_end_ | ri | ST_start_ | ST_end_ | ri | ST_start_ | ST_end_ | ri |
| 1 | 12.9 | 6.6 | 0.49 | 10.9 | 10.6 | 0.02 |  |  |  |
| 3 | 3.3 | 2.8 | 0.14 | 4.0 | 3.1 | 0.24 | 3.5 | 3.3 | 0.05 |
| 4 | 3.6 | 3.0 | 0.16 | 4.6 | 3.8 | 0.17 | 3.2 | 3.0 | 0.08 |
| 6 | 4.9 | 2.9 | 0.41 | 6.3 | 5.0 | 0.21 | 4.4 | 2.3 | 0.47 |
| 7 | 3.7 | 2.5 | 0.34 | 4.3 | 3.2 | 0.25 | 3.8 | 4.0 | -0.04 |
| 8 | 8.6 | 7.7 | 0.10 | 7.8 | 6.3 | 0.20 | 6.9 | 7.7 | -0.12 |
| 9 | 2.1 | 2.0 | 0.01 | 4.4 | 2.9 | 0.33 | 3.0 | 2.5 | 0.19 |
| 10 | 1.4 | 1.1 | 0.22 | 2.2 | 1.6 | 0.27 | 1.7 | 1.4 | 0.21 |
| 11 | 1.5 | 1.2 | 0.20 | 2.1 | 1.6 | 0.24 | 1.7 | 1.5 | 0.09 |
| 12 | 2.6 | 1.9 | 0.25 | 4.6 | 3.1 | 0.31 | 3.3 | 2.6 | 0.23 |
| 13 | 8.1 | 4.5 | 0.45 | 10.6 | 7.0 | 0.34 |  |  |  |
| 14 | 1.4 | 1.2 | 0.17 | 2.4 | 1.7 | 0.30 | 1.6 | 1.5 | 0.07 |
| 15 | 1.6 | 1.6 | 0.00 | 3.5 | 3.0 | 0.14 | 3.0 | 2.7 | 0.10 |
| 16 | 2.9 | 2.2 | 0.25 | 3.1 | 2.6 | 0.17 | 2.8 | 2.4 | 0.17 |
| 17 | 3.4 | 2.4 | 0.29 | 4.6 | 3.3 | 0.29 | 2.6 | 2.2 | 0.16 |
| 19 | 1.8 | 2.0 | -0.09 | 3.1 | 6.0 | -0.94 | 2.4 | 2.0 | 0.15 |
| 20 | 1.5 | 1.2 | 0.18 |  |  |  | 1.6 | 1.4 | 0.12 |
| 22 | 6.4 | 9.0 | -0.41 | 11.8 | 12.9 | -0.10 | 10.8 | 13.5 | -0.25 |
| 25 |  |  |  | 10.4 | 7.2 | 0.31 |  |  |  |
| 28 | 2.6 | 2.2 | 0.14 | 2.7 | 2.7 | 0.00 | 3.1 | 2.6 | 0.14 |
| 30 | 2.0 | 1.6 | 0.18 | 2.4 | 1.9 | 0.23 | 1.9 | 1.6 | 0.18 |
| 31 | 8.6 | 6.8 | 0.22 | 10.0 | 6.2 | 0.38 | 6.8 | 5.2 | 0.24 |
|  | comparison group | | |  |  |  |  |  |  |
|  | ST_T1_ | ST_T2_ | ri |  |  |  |  |  |  |
| 100 | 2.7 | 2.8 | -0.05 |  |  |  |  |  |  |
| 101 | 1.9 | 1.5 | 0.17 |  |  |  |  |  |  |
| 102 | 1.3 | 1.3 | 0.01 |  |  |  |  |  |  |
| 103 | 1.3 | 1.7 | -0.23 |  |  |  |  |  |  |
| 104 | 1.5 | 1.5 | 0.03 |  |  |  |  |  |  |
| 105 | 1.5 | 1.6 | -0.08 |  |  |  |  |  |  |
| 106 | 1.2 | 1.2 | 0.01 |  |  |  |  |  |  |
| 107 | 1.5 | 1.4 | 0.03 |  |  |  |  |  |  |
| 108 | 2.4 | 1.9 | 0.21 |  |  |  |  |  |  |
| 109 | 1.3 | 1.1 | 0.13 |  |  |  |  |  |  |
| 110 | 1.1 | 1.0 | 0.15 |  |  |  |  |  |  |
| 110 | 1.9 | 1.8 | 0.04 |  |  |  |  |  |  |
| 112 | 1.3 | 1.3 | -0.01 |  |  |  |  |  |  |
| 113 | 1.1 | 1.0 | 0.08 |  |  |  |  |  |  |
| 114 | 3.3 | 3.0 | 0.10 |  |  |  |  |  |  |
| 115 | 2.1 | 2.4 | -0.14 |  |  |  |  |  |  |

Missing Data: Patient #25 trained the entire 6 weeks only at level 2. Patient #13 did not train at level 3. In one case (#20), the training data for level 2 were not saved.

The training is available as commercial software VISIO*coach*® Kids (www.visiocoach.de).
