# Supplementary material for: Effects of visual search training in children with hemianopia
Source: PLoS One. 2018 Jul 18;13(7):e0197285. doi: 10.1371/journal.pone.0197285 (PMC6051578; doi:10.1371/journal.pone.0197285)
Supplement: S1 File — (DOC) [file pone.0197285.s002.doc]

**Antrag zur Beurteilung ethischer und rechtlicher Fragen eines medizinischen Forschungsvorhabens am Menschen VERSION 2**

**Hemianopsie bei Kindern: Evaluierung adaptiver Mechanismen als Grundlage für die Rehabilitation sowie Analyse der Struktur-Funktionsbeziehungen zur Prädiktion und Kontrolle der visuellen Funktion bei hirnchirurgischen Eingriffen**

**ANTRAGSTELLERIN:
Prof. Dr. med. Susanne Trauzettel-Klosinski**

**Tel: 07071-298-4831, Fax: 07071-29-5164,**

**E-Mail:** susanne.trauzettel-klosinski@uni-tuebingen.de

**Forschungsstelle:**

Forschungseinheit für Visuelle Rehabilitation

Department für Augenheilkunde, Universität Tübingen

Schleichstr. 12, 72076 Tübingen

**Arbeitsgruppe:**

**Augenklinik Tübingen, Forschungseinheit für Visuelle Rehabilitation**

- Frau Prof. Dr. med. Trauzettel-Klosinski, Leiterin der Forschungseinheit für Visuelle Rehabilitation, Department für Augenheilkunde-Tübingen; Leiterin, Supervisorin und Koordinatorin des Projekts
- Nachwuchswissenschaftler NN
- Frau Cordey und Frau Gehrlich, Orthoptistinnen
- Herr Dr. I. Ivanov, PhD, Physiker und Informatiker, wissenschaftlicher Mitarbeiter
- Frau Krumm, Doktorandin

**Kooperationspartner**

1) Herr Prof. Dr. med. Martin Staudt, Stiftungsprofessur „Neuroplastizität des kindlichen Gehirns“ an der Neuropädiatrie Tübingen; Chefarzt der Klinik für Neuropädiatrie und Neurologische Rehabilitation, Epilepsiezentrum für Kinder und Jugendliche, Schön-Klinik Vogtareuth

2) Herr PD. Dr.med. Marko Wilke: Abteilung für Neuropädiatrie und Entwicklungsneurologie der Universitätsklinik für Kinder- und Jugendmedizin Tübingen:

Kooperationspartner bei der Untersuchung von Kindern mit Hirntumoren

**FACHGEBIET: Augenheilkunde, Neuro-Ophthalmologie, visuelle Rehabilitation, Neuropädiatrie, Neuroplastizität**

**Antragsart : Fortsetzungsantrag**

Für alle verwendeten Untersuchungsmethoden liegen Ethikvoten vor. Der Unterschied zu den früheren Eye Tracking Studien ist das kindliche Alter und kindgerechte Modifikationen der Augenbewegungsmessung und des Trainings:

Für Eye Tracking und Sakkadentraining: 403/2006V und 063/2013BO2 und 433/2011B02.

Für die klinischen Untersuchungsmethoden: 325/2009B01. In diesem Projekt wurden bereits Kinder mit diesen Methoden untersucht.

**FINANZIERUNG:** Technischer Teil Dr. Ivanov: Brunenbusch-Stein-Stiftung, bewilligt

Klinischer Teil beantragt bei der Hannelore Kohl Stiftung

**ANMERKUNG**

Es handelt sich beim beantragten Projekt um die klinische Evaluierung im Rahmen eines großen Projekts. Das Gesamtprojekt besteht aus 3 Teilen, die eng miteinander verzahnt sind:

1) Klinische Evaluierung der visuellen Funktionen und Durchführung des Trainings (das hier beantragte Projekt)

2) Technischer Teil (Eye Tracking, Entwicklung eines kindgerechten Trainingsprogramms) (das hier beantragte Projekt)

3) Bildgebung (MRT, Fiber Tracking) und neuropädiatrische Untersuchung werden von unseren Kooperationspartnern der Neuropädiatrie (s.o.) durchgeführt und finanziert. In der Klinik Vogtareuth (Prof. Staudt) werden die Untersuchungen im Rahmen der stationären Routine-Diagnostik durchgeführt. Dabei liegt der Fokus auf Kindern mit Epilepsie. In der Kinderklinik Tübingen erfolgen die Untersuchungen im Rahmen eines drittmittelfinanzierten Forschungsprojekts mit Fokussierung auf Kinder mit Hirntumoren (PD. Dr. Wilke: „Prä-operative moderne MR-Bildgebung bei Kindern“ – Ethikantrag vom 30.01.14).

Die Daten werden am Ende der Studie zusammengeführt und gemeinsam ausgewertet und publiziert.

**Inhaltsverzeichnis:**

1. Zusammenfassung

2. Einführung

### 2.1 Stand der Forschung

### 2.2 Eigene Vorarbeiten

### 3. Studienziele

### 4. Studienpopulation

4.1. Begründung für die Durchführung der Studie bei Minderjährigen

4.2. Rekrutierung

4.3. Ein- und Ausschlusskriterien

4.4. Studienmedikation

5. Studienablauf und Untersuchungsmethoden

5.1. Studiendesigns

5.2. Durchführung

5.3. Zeitplan

5.4. Untersuchungsmethoden

6. Abbruchkriterien

7. Risiken und Nebenwirkung- Klinische und wissenschaftliche Bewertung

8. Angaben zur statistischen Auswertung

### 9. Klinische und wissenschaftliche Bewertung

10. Datenschutz

11. Aufklärung der Studienteilnehmer

12. Kooperationen

13. Literatur

14. Anlagen

# 1. ZUSAMMENFASSUNG

Die häufigste Ursache für seitengleiche Gesichtsfeldausfälle (homonyme Hemianopsie) bei Kindern ist eine prä- oder perinatal erworbene Hirnschädigung, gefolgt von Schlaganfällen, Hirntumoren oder OP-Folgen, besonders nach epilepsie-chirurgischen Eingriffen. Eine Hemianopsie verursacht eine schwere Behinderung im Alltag, hauptsächlich in Bezug auf die Orientierung im Raum. Es bestehen ein großes Defizit in der spezifischen Diagnostik und ein dringender Bedarf an visuellen Rehabilitationsmaßnahmen bei diesen Kindern.

In dieser Studie besteht jetzt erstmalig die Möglichkeit, erstens: Struktur-Funktions- Beziehungen (Bildgebung vs. Gesichtsfeld); zweitens: die spontanen kompensatorischen Augenbewegungsstrategien bei Kindern mit Hemianopsie unterschiedlicher Genese und Er-krankungsdauer präzise zu untersuchen. Drittens wird die Wirksamkeit und klinische An-wendung einer kompensatorischen Trainingsmethode zur Verbesserung der Orientierung evaluiert und viertens wird bei hirnchirurgischen Eingriffen bei Epilepsie und Hirntumoren die prä- und postoperative Sehfunktion untersucht, um damit in Zukunft bessere Voraussagen über eine operationsbedingte Schädigung treffen zu können, und die Anpassung an die zerebral bedingte Gesichtsfeldschädigung zu erfassen.

Aufgrund der Komplexität der Grunderkrankung und der schwerwiegenden Schädigung der Kinder ist das Forschungsvorhaben nicht nur von großer wissenschaftlicher Bedeutung, sondern auch im Hinblick auf die patientenorientierte Umsetzung, indem Rehabilitationsmaßnahmen spezifisch eingesetzt werden mit dem letztendlichen Ziel, den Kindern Lebenstüchtigkeit und Teilhabe zu ermöglichen.

# 2. Einführung

# 2.1 Hintergrund und Stand der Forschung

Läsionen der Sehbahn oberhalb der Sehnervenkreuzung führen zu seitengleichen (homonymen) Gesichtsfeldausfällen. Sie sind an der vertikalen Mittellinie begrenzt und können je nach Läsionsort eine vollständige Hemianopsie, einen Quadrantenausfall oder einen kleinen parazentralen Ausfall verursachen. Die häufigste Ursache für Hemianopsie bei Kindern ist eine prä- oder perinatal erworbene Hirnschädigung, gefolgt von kindlichen Schlaganfällen, Hirntumoren oder OP-Folgen. Eine besondere Rolle kommt hier den so genannten epilepsie-chirurgischen Eingriffen zu: Wenn Patienten mit Epilepsien nicht ausreichend auf eine medikamentöse Therapie ansprechen, so kommt in manchen Fällen eine chirurgische Entfernung oder Abtrennung (Diskonnektion) der epileptogenen Hirnareale in Frage. Dieser Eingriff führt im Idealfall zur Anfallsfreiheit, birgt jedoch u. a. das Risiko, durch Schädigung funktionstragender Hirnteile entsprechende neurologische Defizite zu hinterlassen. Gesichtsfeldausfälle bis hin zur Hemianopsie sind hierbei eine häufige Problematik; in schweren Fällen wird die Entstehung eines solchen Gesichtsfeldausfalles durch die Operation sogar bewusst in Kauf genommen.

Eine homonyme Hemianopsie verursacht eine schwere Behinderung im Alltag, hauptsächlich in Bezug auf die Orientierung im Raum, wobei die Patienten gegen Gegenstände oder Menschen auf der hemianopen (blinden) Seite stoßen, oder Probleme haben, den Weg richtig zu finden. Daher ist die Rehabilitation bei der hemianopen Orientierungsstörung besonders wichtig.

Frühere Studien haben gezeigt, dass erwachsene Patienten eine spontane Adaptationsstrategie entwickeln können, indem sie spontan Augenbewegungen zur hemianopen Seite machen, um ihr Blickfeld zu vergrößern. Diese spontane Kompensationsstrategie kann durch ein zusätzliches Augenbewegungstraining (Sakkadentraining) verbessert werden (Kerkhoff et al. 1992, Zihl 1995, Pambakian et al. 2000, 2004,Roth et al 2009), siehe unten.

Im Gegensatz zu weit reichenden Erkenntnissen bei Erwachsenen weiß man bislang wenig über die Kompensation von Schädigungen der Sehbahn oder der Sehrinde bei Kindern – weder in Bezug auf neuroplastische Vorgänge als Reaktion auf eine derartige Schädigung noch in Bezug auf adaptive Mechanismen und deren gezielte therapeutisch-rehabilitative Unterstützung. Diesen Fragen soll im vorliegenden Forschungsprojekt nachgegangen werden, um so die Grundlage zu schaffen für eine effektivere Behandlung/Rehabilitation kindlicher Gesichtsfeldausfälle.

# 2.2 Eigene Vorarbeiten

In der Forschungseinheit für Visuelle Rehabilitation mit der angegliederten Sehbehindertenambulanz der Universitäts-Augenklinik Tübingen besteht über eine 20-jährige Erfahrung mit sehbehinderten und blinden Patienten. Frau Prof. Trauzettel-Klosinski hat diese Einrichtung seit 1991 an der Tübinger Universitätsaugenklinik aufgebaut. Die enge Verbindung zwischen Dienstleistung und Forschung hat sich außerordentlich bewährt, da sie eine gute Basis für eine patientennahe Forschung ermöglicht. In unserem Forschungslabor wurden in den letzten 15 Jahren rund 30 über Projektförderung finanzierte Forschungsvorhaben (Gesamtfördervolumen: 3.921475€). Dies hat ermöglicht, daß nicht nur ein kompetentes Mitarbeiterteam aufgebaut werden konnte, sondern auch eine sehr gute apparative Ausstattung besteht.

Im Team der Sehbehindertenambulanz und der Forschungseinheit für Visuelle Rehabilitation arbeiten Augenärzte und Orthoptistinnen. Die interdisziplinäre Zusammenarbeit, die insbesondere für die Kinder mit zerebraler Sehschädigung gefordert ist, wird hier schon seit vielen Jahren erfolgreich praktiziert. Bezüglich der Untersuchung und Versorgung sehbehinderter Kinder mit zusätzlicher Behinderung besteht ebenfalls eine besondere Expertise, die durch ein 3-jähriges von der Hildebrand und Palm Stiftung gefördertes Forschungsprojekt zur Evaluierung visueller Funktionen bei Kindern mit Mehrfachbehinderung erlangt wurde.

Eine besondere Expertise besteht auch in der wissenschaftlichen Analyse und Rehabilitation bei Orientierungs- und Lesestörungen (TK siehe Literaturverzeichnis). Unsere Arbeitsgruppe hat wir in den letzten Jahren eine evidenz-basierte Trainingsmethode entwickelt, bei Erwachsenen-Patienten mit Hemianopsie nach Schlaganfall angewendet wurden (Roth et al 2009a,b;Trauzettel-Klosinski 2009). Diese Studie wurde 2009 mit dem Fürst-Donnersmark Forschungspreis für neurologische Rehabilitation ausgezeichnet. Diese Studie zeigte, dass die Orientierungsfähigkeit und die Lebensqualität durch ein gezieltes Augenbewegungstraining (Sakkadentraining) deutlich und nachhaltig verbessert werden kann. Bei den o.g. Patienten wurden mit Hilfe einer Suchaufgabe die Augenbewegungen zum blinden Halbfeld hin gezielt trainiert, um das blinde Halbfeld besser abzuscannen und durch zusätzliche Aufmerksamkeitsverschiebung das Blickfeld zu erweitern. Obwohl der mit dem Perimeter gemessene Halbseitengesichtsfeldausfall unverändert blieb, konnten die Patienten durch die neu erlernte Strategie der Augenbewegungen Personen und Objekte im freien Raum besser finden.

Die Abteilung Neuropädiatrie und Entwicklungsneurologie (Ärztliche Direktorin: Prof. Ingeborg Krägeloh-Mann) der Universitätsklinik für Kinder- und Jugendmedizin beschäftigt sich seit vielen Jahren mit frühen Hirnläsionen, und zwar bezüglich Entstehung, Auswirkungen und Möglichkeiten der Kompensation und Reorganisation. Neben der motorischen Domäne (Zerebralparese) treten zunehmend auch somatosensorische, sprachliche und kognitive Aspekte in den Vordergrund.

Der Haupt-Kooperationspartner Prof. M. Staudt geht seit 1999 Fragen der Reorganisation nach frühen Hirnläsionen nach, im Rahmen mehrerer Forschungsprojekte. Ein Schwerpunkt war dabei die multimodale Erfassung von reorganisierten sensomotorischen Systemen, bei denen die Methoden der strukturellen und funktionellen MRT, des Diffusion Tensor Imaging mit Fiber Tracking, der transkraniellen Magnetstimulation und der Magnetenzephalographie zum Einsatz kamen. Aktuell bekleidet Prof. Staudt zwei Positionen: Er ist Stiftungsprofessor für „Neuroplastizität des kindlichen Gehirns“ an der Abteilung Neuropädiatrie und Entwicklungsneurologie der Universitätsklinik für Kinder- und Jugendmedizin Tübingen und gleichzeitig Chefarzt der Klinik für Neuropädiatrie und Neurologische Rehabilitation, Epilepsiezentrum für Kinder und Jugendliche der Schön-Klinik Vogtareuth. Diese Doppelfunktion schafft ideale Voraussetzungen für die Durchführung des vorgelegten Forschungsvorhabens, weil die methodische und wissenschaftliche Expertise der Universität (Augenklinik und Kinderklinik) kombiniert werden kann mit dem hohen Aufkommen an geeigneten Patienten (sowohl aus der Epilepsiechirurgie als auch aus der Neurorehabilitation) der Schön Klinik Vogtareuth.

Die Schön Klinik Vogtareuth ist ein hochspezialisiertes Krankenhaus für Neuropädiatrie, Neurologie und Rehabilitationsmedizin sowie chirurgische Fachdisziplinen. Besonders in der Neuropädiatrie, der Neurochirurgie und insbesondere der Epilepsiebehandlung von Kindern und Jugendlichen hat die Schön Klinik Vogtareuth einen weit überregionalen Einzugsbereich. So werden pro Jahr 40-50 epilepsiechirurgische Eingriffe bei Kindern und Jugendlichen durchgeführt. Die Besonderheit sowie die Stärke der Klinik ist die Kombination eines Epilepsiezentrums für Kinder und Jugendliche mit einer pädiatrischen Neurorehabilitationsklinik, was eine ganzheitliche Betreuung "unter einem Dach" sowie einen intensiven Austausch zwischen den beiden Disziplinen ermöglicht.

Der zweite Kooperationspartner, PD. Dr. Wilke, leitet den interdisziplinären Arbeitsbereich „Experimentelle Pädiatrische Neurobildgebung“ an der Abteilung Neuropädiatrie und Entwicklungsneurologie der Universitätsklinik für Kinder- und Jugendmedizin. Auf der Basis umfangreicher Vorarbeiten wurde kürzlich ein Projekt begonnen mit dem Titel „Prä-operative moderne MR-Bildgebung bei Kindern“. Unsere Kooperation besteht darin, dass wir die visuelle Funktion von Kindern mit Hirntumoren prä- und postoperativ untersuchen, eine Struktur-Funktionsbeziehung (Bildgebung versus Gesichtsfeld) ermitteln und damit die Prädiktion von postoperativen Gesichtsfeldausfällen verbessern. Außerdem soll auch bei diesen Kindern die spontane Anpassung an den akut aufgetretenen Ausfall untersucht sowie das Angebot einer Rehabilitation mit einem Sakkadentraining gemacht werden.

### 3. Studienziele

Im Vergleich zwischen Kindern mit prä- oder perinatal erworbenen Läsionen (Kollektiv a) und Kindern mit spät (jenseits des zweiten Lebensjahres) erworbenen Läsionen (Kollektiv b) sollen folgende Ziele verfolgt werden:

1. Etablierung von Struktur-Funktions-Beziehungen zwischen Läsionen von Sehbahn und / oder Sehrinde und dem Gesichtsfeldausfall

Hypothese: Vergleichbare Läsionen verursachen umso geringere Ausfälle, je früher sie entstehen

2. Erfassung der spontanen adaptiven Mechanismen bei Kindern mit Hemianopsie

Hypothese: je früher die Läsion entsteht, umso ausgeprägter / effektiver sind die Kompensationsmechanismen

3. Evaluierung einer (im Rahmen des technischen Projekts bis Studienbeginn entwickelten) kindgerechten Modifizierung / Optimierung des für Erwachsene etablierten Sakkaden-Trainingsprogramms bei Kindern mit Hemianopsie

4. bei Kindern mit akut induzierten Hemianopsien (im Rahmen epilepsie-chirurgischer Eingriffe oder nach Hirntumor-Operationen) der zeitliche Verlauf des Gesichtsfeldausfalls und der Entstehung von Kompensationsmechanismen verfolgt werden (Kollektiv c).

# 4. Studienpopulation

Kollektiv a: n = 30 Kinder mit prä- oder perinatal erworbenen Läsionen von Sehbahn und / oder Sehrinde

Kollektiv b: n = 10 Kinder mit später (jenseits des 2. Lebensjahres) erworbenen Läsionen von Sehbahn und / oder Sehrinde – mindestens schon ein Jahr bestehend.

Kollektiv c: je nach Patientenanfall - möglichst viele (ca. 4 pro Jahr) Kinder, die iatrogen im Rahmen epilepsiechirurgischer Eingriffe akut und „geplant“ einen Gesichtsfeldausfall entwickeln (Resektionen oder Diskonnektionen eines Okzipitallappens, Hemisphärektomien). Hinzu kommen Kinder bei akut aufgetretener Hemianopsie nach Hirntumor-Operationen (voraussichtlich 3-4 pro Jahr). Die Besonderheit dieser Unterstudie liegt an der Seltenheit der beiden Erkrankungen, welche eine möglichst genaue Evaluierung des spontanen Verlaufes der zerebral bedingten Gesichtsfeldschädigung mit dem Ausblick auf ein in Zukunft mögliche, rechtzeitig eingesetzte Trainingsmethode erlaubt.

Kollektiv d: Bei 10 gesunden altersangepassten Kontrollkindern sollen Normalwerte für das Scanningverhalten ermittelt werden.

## 4.1. Begründung, warum die Studie an Minderjährigen durchgeführt werden muss:

Die häufigste Ursache für Hemianopsie bei Kindern ist eine prä- oder perinatal erworbene Hirnschädigung, gefolgt von kindlichen Schlaganfällen, Hirntumoren oder OP-Folgen, besonders nach epilepsie-chirurgischen Eingriffen. Die Epilepsien gehören mit einer Inzidenz von 0,6 Promille und einer Prävalenz von 0,5 Promille zu den häufigsten chronischen Erkrankungen im Kindesalter bis zu 10 Jahren.

Da über die Struktur-Funktionsbeziehungen (MRT vs. Gesichtsfeld) bei kindlichen Hirnläsionen bisher wenig bekannt ist und diese Kinder bisher unzureichende Rehabilitationsangebote haben, ist diese Studie nur an Kindern durchführbar.

## 4.2 Rekrutierung

Die Rekrutierung der Patienten erfolgt hauptsächlich in der Schön Klinik Vogtareuth.

Die Rekrutierung der Kinder mit Hirntumoren erfolgt in der Neuropädiatrie Tübingen, Herrn PD Dr.Wilke, unserem direkten Kooperationspartner. Die Operationen erfolgen in der Neurochirurgischen Abteilung Tübingen.

Die Rekrutierung der Kontrollkinder erfolgt aus dem Bekanntenkreis der Mitarbeiter der Forschungseinheit für Visuelle Rehabilitation. Auf die Freiwilligkeit der Teilnahme wird besonders hingewiesen.

**4.3. Ein- und Ausschlusskriterien**

Einschlusskriterien

- Alter 6-18 Jahre, bevorzugt ab 10 Jahre

- Vorliegen von neuro-pädiatrischen Befunden
- Vorliegen von MRT-Befunden
- Einwilligung von mindestens einem Elternteil

Ausschlusskriterien

- mangelnde Kooperationsfähigkeit

# 4.4. Studienmedikation

In der Studie werden keine Medikamente getestet. Eine Erweiterung der Pupille und eine Zykloplegie kann bei einigen Kindern notwendig sein (siehe Kapitel 7).

# 5. Studienablauf und Untersuchungsmethoden

5.1 Studiendesigns

Das beantragte Forschungsprojekt ist eine prospektive, klinische Diagnostik- und Interventionsstudie, die mit einem labortechnischen Projekt für die Entwicklung von Untersuchung- und Rehabilitationsmethoden bei Kindern mit Hemianopsie eng verknüpft ist (siehe Flowchart). Die technischen Vorarbeiten sind bereits im Gang. Alle Untersuchungen und Maßnahmen werden in einer intensiven Kooperation zwischen der Forschungseinheit für Visuelle Rehabilitation des Departments für Augenheilkunde Tübingen, der Abteilung Neuropädiatrie, Entwicklungsneurologie, SPZ der Universitätsklinik für Kinder- und Jugendmedizin Tübingen und Abteilung für Neuropädiatrie, Neurologische Rehabilitation und Epilepsie (Kinder und Jugendliche) der Schön Klinik Vogtareuth durchgeführt. Die Vorteile eines solchen interdisziplinären Forschungsvorhabens bestehen darin, dass einzelne, qualitativ hochwertige Leistungen (ophthalmologisch und neuropädiatrisch) koordiniert werden, damit die Kinder auf diese Weise eine optimale Diagnostik und Rehabilitationsmaßnahmen erhalten.

- 1. Durchführung

Die Untersuchungen werden in der Forschungseinheit für Visuelle Rehabilitation des Departments für Augenheilkunde der Universität Tübingen im ViTa –Gebäude Osianderstr. 5 durchgeführt.

- 1. Zeitplan

Das Projekt besteht aus einem klinischen Teil und einem technischen Teil, der die Studie technisch unterstützt und mit einem Vorlauf von 7 Monaten bereits entsprechende Vorarbeiten durchgeführt hat. Dadurch kann das klinische Projekt direkt mit der Pilotstudie beginnen (siehe Graphik).

Technische Vorarbeiten

Pilotstudie

Pilotstudie

Hauptstudie

Daten-analyse Publikation

techn. Teil

klin. Teil

7 Monate

14 Monate

3 Monate 1.8.-31.10.14

1.7.-30.9.14

1

18 Monate

1.11.14 – 31.4.16

3 Monate

1.5.16 – 31.07.16

1. Jahr

2. Jahr

Eye Tracking, Software-Weiter-Entwicklung, MRT-Analyse

**5.4. Untersuchungsmethoden**

Alle Untersuchungen werden ambulant durchgeführt. Das komplette Untersuchungsprotokoll dauert ca.3 Stunden pro Patient mit zusätzlichen Pausen.

1. **Ermittlung der Sehschärfe für die Ferne und für die Nähe bei standardisiertem Beleuchtungsniveau** unter Einsatz von unterschiedlichen Sehtests je nach Alter und nach Schweregrad der klinischen Befunde sowie der Kooperationsfähigkeit und. Einbezogen ist die objektive Refraktionsbestimmung mittels Skiaskopie oder Refraktometer.
2. **Bestimmung des Kontrastsehens mit Lea-Zahlen oder - Bildern bei 10% Kontrast.**
3. **Orthoptischer Status**: Fixation, Augenstellung, Motilität, Binokularsehen, Dominanz
4. **Gesichtfelduntersuchung**: im Hinblick auf die alltagsrelevanten Tätigkeiten wie Naharbeit, Lesen und Orientierung wird hier speziell das zentrale 30° Gesichtsfeld untersucht, wenn möglich mit dem Tübinger Handperimeter. Stark behinderte Kinder sind nicht in der Lage, bei einer herkömmlichen Perimetrie mitzuarbeiten. Bei diesen wird dann eine Tangent Screen Kampimetrie durchgeführt, bei der die weißen Stimuli manuell auf einem schwarzen Hintergrund an einer Wand angeboten werden. Noch weniger Mitarbeit verlangt die von uns entwickelte LED-Kampimetrie, bei der Blickzielbewegungen zum Reiz erlaubt sind. Im 30° Gesichtsfeld werden Lichtreize in Form von weißen Leuchtdioden dargeboten. Damit handelt es sich im engeren Sinne um eine Messung des Blickfeldes. Der Test nützt eine reflektorische Reaktion als Antwort und ist nicht auf subjektive Angaben angewiesen. Alle anderen Untersuchungsmethoden wurden bereits in früheren Studien mit positiven Ethikvoten eingesetzt (s.S. 1 unten) – außer Punkt 6 (siehe dort) . Alle Untersuchungen werden nur von qualifizierten Mitarbeitern durchgeführt. Sämtliche Geräte werden nur ihrer Zweckbestimmung entsprechend verwendet und instand gehalten.
5. **Klinisch-morphologische Untersuchung der vorderen und hinteren Augenabschnitte** mit genauer Erhebung des ophthalmologischen Befundes.
6. **Augenbewegungen** während des Betrachtens einer natürlichen Szene am Bildschirm sowie während Suchaufgaben mittels eines Infrarot Eye Trackers (Saccadometer, Jazz novo, Ober). Die Messeinheit befindet sich an der Nasenwurzel und beleuchtet tangential den Limbus. Sie wird mit einem elastischen Band am Kopf befestigt. Das System hat damit vor allem für Kinder den Vorteil, nicht direkt vor den Augen zu sitzen. Bei der Erstuntersuchung werden die spontanen Anpassungsstrategien erfasst, nach dem Training die potentielle Verbesserung. Eine in-house Zertifizierung wird zur Zeit durchgeführt. Die Bescheinigung wird nachgereicht.

1. **Sakkadentraining** mit einer kindgerechten Software bestehend aus einer Suchaufgabe am Bildschirm: Das Kind erhält eine Einweisung bei uns und kann dann zu Hause am eigenen PC selbständig üben: 2x15 min/Tag an 5 Tagen/Woche für 6 Wochen. Sollte kein eigener PC vorhanden sein, erhält das Kind von uns einen Laptop leihweise.

### 6. Abbruchkriterien

Die Studienteilnahme kann von den Patienten jederzeit ohne Angabe von Gründen folgenlos abgebrochen werden. Zwingende Abbruchkriterien sind zudem eine starke Überforderung oder Ermüdung des Patienten.

### 7. Risiken und Nebenwirkungen

Die im Rahmen der Studie durchgeführten ophthalmologischen Untersuchungen entsprechen hinsichtlich Risiken und möglicher Nebenwirkungen denen einer augenärztlichen Routineuntersuchung. Die meisten Untersuchungsmethoden sind standardisiert und bereits in der Klinik routinemäßig eingesetzt worden.

Für die Untersuchung des Augenhintergrundes ist i.d.R. eine Weitstellung der Pupillen mit einem kombinierten Parasympatholytikum und Sympathomimetikum erforderlich. Zur Pupillenerweiterung verwenden wir das parasympatholytische Medikament Tropicamid topisch, das auch in der Routine augenärztlicher Untersuchungen eingesetzt wird. Die Gefahr eines Glaukomanfalls durch die Pupillenerweiterung ist bei Kindern kaum vorhanden.

Zur objektiven Refraktionsbestimmung ist bei jüngeren Kindern manchmal eine Cycloplegie erforderlich. Dazu verabreichen wir Augentropfen, die den Wirkstoff Cyclopentolathydrochlorid (Cyclopentolat 1%, oder 0,5%) enthalten und i.d.R. 2-3x im Abstand von 10 Minuten getropft werden. Wir bitten zuvor die Neuropädiater um kurze schriftliche Empfehlung, welche Augentropfen wir verabreichen dürfen.

Die Pupillenerweiterung hält für ca. 3-4 Stunden an. Während dieser Zeit, kann es zu einer verstärkten Lichtempfindlichkeit und einer Einschränkung des Sehvermögens in der Nähe kommen.

Die von uns entwickelte LED-Kampimetrie verwendet nur Leuchtdioden mit den EU-Sicherheitskennzeichen (bereits in früherer Studie eingesetzt).

Für das Training und die Suchaufgaben werden handelsübliche Computer eingesetzt, die entsprechend der geltenden Vorschriften strahlungsarm sind (bereits in früheren Studien eingesetzt).

Der hier verwendete Infrarot Eye Tracker ist besonders gut für Kinder geeignet, da er nur mit einem Kopfband befestigt ist und die Messeinheit nicht direkt vor den Augen sitzt. Andere Infrarot Eye Tracker haben wir bereits in früheren Studien eingesetzt.

### 8. Klinische und wissenschaftliche Bewertung

Die Studie ist nicht nur von innovativer wissenschaftlicher Bedeutung, sondern auch sehr patientenorientiert. Das Training kann den Kindern einen Benefit bereiten für die Bewältigung von alltagsrelevanten Aufgaben.

# 9. Angaben zur statistischen Auswertung

Die Daten der ophthalmologischen Untersuchungen werden bei Patienten getrennt von den neuropädiatrischen und MRT Untersuchungen erhoben und in eine Datenbank eingegeben und dann anhand der pseudonymisierten Zuordnung mit den klinisch-neurologischen und kernspintomographisch-strukturellen Befunden verglichen.

Der Schwerpunkt soll auf der Korrelation zwischen ophthalmologischen, neuropädiatrischen und kernspintomographisch-strukturellen Befunden liegen.

# 10. Datenschutz

Die Daten werden auf Fragebögen bzw. Untersuchungsbögen und in Computerdateien erfasst. Die Daten werden direkt nach der Erfassung in pseudonymisierter Form gespeichert. Dazu wird ein Code zugeteilt und eine Liste geführt, die eine Rückführung der Daten ermöglichen würde.

Die Kodierungsunterlagen und die Kodierungsliste werden zusammen mit den Einverständniserklärungen in einem verschlossenen Schrank aufbewahrt, zu dem nur die Prüfärzte Zugang haben. Sie wird Dritten nur in den in der Einwilligungserklärung zum Datenschutz genannten Fällen zugänglich gemacht. Die Daten werden 10 Jahre aufbewahrt.

# 11. Aufklärung der Studienteilnehmer

Jeder Proband und jeder Patient/in mit zumindest einem Elternteil werden vor ihrer Einwilligung durch den Untersuchungsleiter ausführlich über den Ablauf der Untersuchung informiert. Alle Probanden/Patienten und deren Eltern werden ausdrücklich darauf hingewiesen, dass sie ihre Teilnahme, bzw. die Teilnahme ihres Kindes, an der Studie jederzeit und ohne Angabe von Gründen beenden können. Erst danach geben die Untersuchungsteilnehmer und deren Eltern ihre Zustimmung zu den Untersuchungen.

Bei den Kindern mit hirnchirurgischen Eingriffen werden die Eltern im Rahmen der postoperativen Vorstellung, falls sich ein Defizit zeigt, über die mögliche Teilnahme an einer Folgestudie (Sakkadentraining)aufgeklärt, bei der die Rehabilitation und Reorganisation gemeinsam mit Herrn Professor Staudt untersucht werden.

Vergütung: Die Kinder erhalten nach Abschluss der letzten Untersuchung einen Büchergutschein im Wert von 25.-€.

# 12. Kooperationen (Einzelheiten siehe Seite 1)

**Schön-Klinik Vogtareuth**

- Herr Prof. Dr. med. Martin Staudt, Chefarzt der Klinik für Neuropädiatrie und Neurologische Rehabilitation, Epilepsiezentrum für Kinder und Jugendliche, Schön-Klinik Vogtareuth

**Abteilung Neuropädiatrie Tübingen**

- Herr PD. Dr.med. Marko Wilke: Kooperationspartner bei der Untersuchung von Kindern mit Hirntumoren
- Herr Prof. Dr. med. Martin Staudt, Stiftungsprofessur „Neuroplastizität des kindlichen Gehirns“

Tübingen, den 24.07.2014

Prof. Dr. med. Susanne Trauzettel-Klosinski

**13. Literatur**

**LITERATUR ZUM THEMA**

**Literatur anderer Autoren**

- Ahmed M, Dutton GN (1996). Cognitive visual dysfunction in a child with cerebral damage. Developmental Medicine and Child Neurology 38: 736-743
- Baker-Nobles L, Rutherford A (1995). Understanding cortical visual impairment in children. American Journal of occupational Therapy 49: 899-903
- Barkovich AJ, (1992). MR and CT evaluation of the profound neonatal and infantile asphyxia. Am J Neuroradiol 13: 959-972
- Buonomano DV, Merzenich MM (1998) Cortical plasticity: From synapses to maps. Avv Rev Neurosci 2: 149-186
- Dutton GN, Ballantyne J, Boyd G. Bradnam M, Day R, McCulloch D, Mackie R, Phillips S, Saunders K (1996). Cortical visual dysfunction in children: a clinical study. Eye 10: 302-309
- Dutton GN, Jacobson LK (2001). Cerebral visual impairment in children. Semin Neonatol. Dec; 6 (6):477-85. Review
- Good WV, Jan JE, Burden SK, Skoczenski A, Candy R. Recent advances in cortical visual impairment (2001). Dev Med Child Neurol. Jan;43(1): 56-60. Review
- Good WV, Jan JE, deSa L, Barkovich KAJ, Groenveld M, Hoyt CS (1994). Cortical visual impairment in children: a major review. Survey of Ophthalmology 88: 351-364
- Groenveld M, Jan JE, Leader E (1990). Observations on the habilitation of children with cortical visual impairment. Journal of Visual Impairment and Blindness 84: 11-15
- Guzzetta A, Mercuri E, Cioni G (2001). Visual disorders in children with brain lesions: 2. Visual impairment associated with cerebral palsy. Eur J Paediatr Neurol. 5(3): 115-9, Review
- Guzzetta A, D'Acunto G, Rose S, Tinelli F, Boyd R, Cioni G. Plasticity of the visual system after early brain damage. Dev Med Child Neurol. 2010 Oct;52(10):891-900. doi: 10.1111/j.1469-8749.2010.03710.x. Epub 2010 Jun 15. Review.
- Guzzetta A, Fiori S, Scelfo D, Conti E, Bancale A. Reorganization of visual fields after periventricular haemorrhagic infarction: potentials and limitations. Dev Med Child Neurol. 2013 Nov;55 Suppl 4:23-6. doi: 10.1111/dmcn.12302. Review.
- Hoyt CS (2003). Visual function in the brain-damaged child. Eye. Apr; 17(3): 369-84, Review
- Hoyt CS (2007). Brain injury and the eye. Eye 21: 1285-1289
- Jacobson L, Flodmark O, Martin L. Visual field defects in prematurely born patients with white matter damage of immaturity: a multiple-case study. Acta Ophthalmol Scand. 2006 Jun;84(3):357-62.
- Jacobson L, Rydberg A, Eliasson AC, Kits A, Flodmark O. Visual field function in school-aged children with spastic unilateral cerebral palsy related to different patterns of brain damage. Dev Med Child Neurol. 2010 Aug;52(8):e184-7. doi: 10.1111/j.1469-8749.2010.03650.x. Epub 2010 Apr 30
- Kerkhoff, G., Münßinger, U. Haaf, E. Eberle-Strauss, G., Stögerer, E. (1992): Rehabilitation of homonymous scotomata in patients with postgeniculate damage of the visual system: saccadic compensation training. Restor neurol Neurosci 4: 245-254
- Kommerell, G., Lieb, B., Münßinger, U.(1999): Rehabilitation bei homonymer Hemianopsie. Z prakt Augenheilkunde 20: 344-352
- Pambakian, A.L., Mannan, S.K., Hodgson, T.L., Kennard, C. (2004) Saccadic visual search training: a treatment for patients with homonymous hemianopia. J Neurol Neurosurg Psychiatry 75, 1443–1448
- Pambakian, A.L., Wooding, D.S., Patel, N., Morland, A.B., Kennard, C.,
  Mannan, S.K. (2000) Scanning the visual world: a study of patients with homonymous hemianopia. J Neurol Neurosurg Psychiatry 69, 751–759
- Tinelli F, Guzzetta A, Bertini C, Ricci D, Mercuri E, Ladavas E, Cioni G. Greater sparing of visual search abilities in children after congenital rather than acquired focal brain damage. Neurorehabil Neural Repair. 2011 Oct;25(8):721-8. doi: 10.1177/1545968311407780. Epub 2011 Jun 6.
- Zihl, J.(1995): Visual scanning behaviour in patients with homonymous hemianopia. Neuropsychologia 33: 287-303

**Eigene Publikationen zum Thema**

- Trauzettel - Klosinski S (1997) Eccentric fixation in hemianopic field defects - a valuable strategy to improve reading ability and an indication for cortical plasticity. Neuro - Ophthalmol 18: 117-131
- Trauzettel - Klosinski S, Brendler K (1998) Eye movements in reading with hemianopic field defects: the significance of clinical parameters. Graefe`s Arch Clin Exp Ophthalmol 236: 91-102
- Trauzettel - Klosinski S, Reinhard J (1998) The vertical field border in human hemianopia and its significance for fixation behavior and reading. Invest Ophthalmol Vis Sci 39. 2177-2186
- *Atwell Award und Fortune Poster-Preis -*
- Trauzettel-Klosinski S (2004) Rehabilitation bei homonymer Hemianopsie - ein Überblick. Z prakt Augenheilk 25: 298-304
- Reinhard J, Schreiber A, Vonthein R, Schiefer U, Trauzettel-Klosinski S (2004) Visuelles Restitutionstraining bei homonymer Hemianopsie. Z prakt Augenheilk 25: 305-312
- Reinhard J, Schreiber A, Schiefer U, Sabel BA, Kasten E, Kenkel S, Vonthein R, Trauzettel-Klosinski S (2005) Does visual restitution training change absolute homonymous scotoma? Brit J Ophthalmol 89: 30-35
- Roth T, Sokolov AN, Messias A, Roth P, Weller M, Trauzettel-Klosinski S. (2009) Comparing explorative saccade and flicker training in hemianopia: a randomized controlled study. Neurology 72(4): 324-331.
- *Fürst Donnersmarck Forschungspreis für Neurorehabilitation -*
- Roth T, Sokolov A, Messias A, Roth P, Weller M, Trauzettel-Klosinski S (2009) Sakkadentraining verbessert visuelle Exploration bei Hemianopsie – Eine randomisierte kontrollierte Studie. Z prakt Augenheilk 30:403-410
- Trauzettel-Klosinski S (2009) Rehabilitation bei Sehbahnschäden. Klin Monatsbl Augenheilk 226: 897-907
- Trauzettel-Klosinski S (2010) Rehabilitation for Visual Disorders. J Neuro-Ophthalmol 30: 73-84
- Trauzettel-Klosinski S (2011) Zeitgemäße Möglichkeiten visueller Rehabilitation -

Up-to date Options for Visual Rehabilitation. Dtsch Aerztebl 108, 51/52, 871-878

- Cordey A, Trauzettel-Klosinski S (2012) Exploratives Sakkadentraining – Kompensation von homonymen Gesichtsfelddefekten. Orthoptik - Pleoptik 35: 19-28
- Trauzettel-Klosinski S (2012) Visuelles Rehabilitationstraining bei homonymen Gesichtsfeldausfällen. Ophthalmologe, 109:496 - 500 / DOI 10.1007/s00347-012-2571-6
- Trauzettel-Klosinski S, Dietz K and the IReST Study Group (2012) Standardized Assessment of Reading Performance: The new International Standardized Reading Texts IReST. Invest Ophthalmol Vis Sci 53:5452-5461
- Reinhard J, Damm I, Ivanov IV, Trauzettel-Klosinski S (2014) Eye movements during saccadic and fixation tasks in patients with hemianopia. J Neuro-Ophthalmol, in print

**Literatur der Kooperationspartner zum Thema**

- Pascoal T, Paglioli E, Palmini A, Menezes R, Staudt M: Immediate improvement of motor function after epilepsy surgery in congenital hemiparesis. Epilepsia. 2013; 54(8): e109-111
- van der Kolk N, Boshuisen K, van Empelen R, Koudijs S, Staudt M, van Rijen P, van Nieuwenhuizen O, Braun K: Etiology-specific differences in motor function after hemispherectomy. Epilepsy Res 2013 Feb;103(2-3):221-30
- Zsoter A, Pieper T, Kudernatsch M, Wilke M, Staudt M: Predicting hand function after hemispherotomy: TMS versus fMRI in hemispheric polymicrogyria. Epilepsia 2012, 53(6):e98-e101
- Juenger H, DeHaan B, Krägeloh-Mann I, Staudt M, Karnath HO: Early determination of somatosensory cortex in the human brain. Cerebr Cortex 2011;21(8):1827-31

*– Desitin-Jungforscherpreis der Deutschen Gesellschaft für Neuropädiatrie 2011 –*

- Staudt M: Reorganization after pre- and perinatal brain lesions. Journal of Anatomy; Special Edition: Development of the Neocortex. 217(4):469-474 (2010) [invited review]
- Wilke M, Staudt M, Juenger H, Grodd W, Braun C, Krägeloh-Mann I: Somatosensory system in two types of motor reorganization in congenital hemiparesis: topography & function. Hum Brain Map, 2009; 30: 776-788
- Staudt M, Ticini LF, Grodd W, Krägeloh-Mann I: Functional topography of early periventricular brain lesions in relation to cytoarchitectonic probabilistic maps. Brain Lang 2008; 106:177-83.
- Staudt M: (Re-)organization of the developing human brain following periventricular white matter lesions. Neurosci Biobehav R 31 (2007) 1150–1156
- Staudt M, Braun C, Gerloff C, Erb M, Grodd W, Krägeloh-Mann I: Developing somatosensory projections bypass periventricular brain lesions. Neurology 2006;67: 522-525
- Staudt M, Erb M, Braun C, Gerloff C, Grodd G, Krägeloh-Mann I: Extensive perlesional connectivity in congenital hemiparesis. Neurology 66: 771 (2006)
- Staudt M, Gerloff C, Grodd W, Holthausen H, Niemann G, Krägeloh-Mann I: Reorganization in congenital hemiparesis acquired at different gestational ages. Ann Neurol, 56 (2004): 854-863

*– Gayle G. Arnold Award for Excellence in the Care of Children with Cerebral Palsy
 der American Academy for Cerebral Palsy and Developmental Medicine AACPDM–*

- Staudt M, Grodd W, Gerloff C, Erb M, Stitz J, Krägeloh-Mann I: Two types of ipsilateral reorganization in congenital hemiparesis: a TMS and fMRI study. Brain 125 (2002):2222-37

*– Desitin-Jungforscherpreis der Deutschen Gesellschaft für Neuropädiatrie 2003 –*

- Staudt M, Grodd W, Niemann G, Wildgruber D, Erb M, Krägeloh-Mann I: Early left periventricular brain lesions induce right hemispheric organization of speech. Neurology 57 (2001): 122-125

*– Alois-Kornmüller-Preis der Deutschen Gesellschaft für Klinische Neurophysiologie –*

- Wilke M, Pieper T, Lindner K, Dushe T, Staudt M, Grodd W, Holthausen H, Krägeloh-Mann I (2011): Clinical functional MRI of the language domain in children with epilepsy. Hum Brain Mapp 32: 1882-1893
- Zsoter A, Staudt M, Wilke M (2012). Identification of successful clinical fMRI sessions in children: an objective approach. Neuropediatrics 43: 249-57

**14. Anlagen**

# Kurzinformation zur Studie für Kinder aus Vogtareuth

# Kurzinformation zur Studie für Kinder mit Hirntumoren aus Tübingen

# Ausführliche Information zur Studie für Kinder aus Vogtareuth

# Ausführliche Information zur Studie für Kinder mit Hirntumoren aus Tübingen

# Einverständniserklärung der Patienten/Eltern
