# Supplementary material for: Effects of visual search training in children with hemianopia
Source: PLoS One. 2018 Jul 18;13(7):e0197285. doi: 10.1371/journal.pone.0197285 (PMC6051578; doi:10.1371/journal.pone.0197285)
Supplement: S2 File — (DOC) [file pone.0197285.s003.doc]

**Application for the assessment of ethical and legal questions concerning a medical research project in humans VERSION 2**

**Hemianopia in children: evaluation of adaptive mechanisms as a basis for rehabilitation and for analysis of structure-function relations for the prediction and control of visual functions in brain surgery**

**APPLICANT: Prof. Susanne Trauzettel-Klosinski, MD**

Tel: 07071-298-4831, Fax: 07071-29-5164,

E-Mail: susanne.trauzettel-klosinski@uni-tuebingen.de

**Research Center:**

Vision Rehabilitation Research Unit

Centre for Ophthalmology

University of Tuebingen

Schleichstr. 12, 72076 Tübingen, Germany

**Working Group:**

Vision Rehabilitation Research Unit, Centre for Ophthalmology at the University of Tuebingen

• Prof. Dr. med. Trauzettel-Klosinski, Head of Vision Rehabilitation Research Unit, Centre for Ophthalmology University of Tuebingen; Principal investigator, supervisor and coordinator of the project

• Junior scientist NN

• Mrs. Cordey and Mrs. Gehrlich, orthoptists

• I. Ivanov, PhD, physicist and computer scientist, research assistant

• Mrs. Krumm, doctoral student

**Cooperation partners**

1) Prof. Dr. med. Martin Staudt, Founding professor "Neuroplasticity of the child's brain" at Neuropadiatrics, Tübingen; Chief Physician of the Department of Neurophysiology and Neurological Rehabilitation, Epilepsy Center for Children and Adolescents, Schön-Klinik Vogtareuth

2) PD. Dr. med. Marko Wilke: Experimental Pediatric Neuroimaging University Children's Hospital Dept. III (Pediatric Neurology):

Co-operation partner in the investigation of children with brain tumors

**Area of expertise: Ophthalmology, neuro-ophthalmology, visual rehabilitation, neuro-pediatrics, neuroplasticity**

**Type of request: Renewal proposal**

For all test and examination methods, positive ethics committee votes have been achieved in previous projects. The differences relative toearlier eye tracking studies are: childhood age, modifications of the eye movement measurement, and the training to suit the needs of children with hemianopia.

For eye tracking and saccadic training: 403 / 2006V, 063 / 2013BO2, and 433 / 2011B02.

For clinical examination methods: 325 / 2009B01. In this project, the children were already examined using these methods.

**FINANCING**: Technical section Dr. Ivanov: Brunenbusch-Stein Foundation, approved

Clinical part applied for at the Hannelore Kohl Stiftung

**REMARK**

The proposed project is the clinical evaluation within a major project. The overall project consists of 3 parts that are closely interlinked:

1) Clinical evaluation of visual functions and training

(the project submitted here)

2) Technical part (eye tracking, development of a training program suitable for children

(the project submitted here)

3) Imaging (MRI, fiber tracking) and neuropediatric examination are carried out and financed by our cooperation partners of the Dept. of Neuropediatrics (s.o.). In the Vogtareuth Clinic (Prof. Staudt), the examinations are carried out within the realm of routine diagnostics. The focus is on children with epilepsy. In the pediatric hospital Tuebingen the examinations are carried out as part of a third-party funded research project focusing on children with brain tumor (PD Dr. Wilke)

"Pre-operative modern MR imaging in children" - Ethics application of 30.01.14).

The data will be collected at the end of the study and analyzed and published together.

Table of Contents:

1. Summary

2. Introduction

2.1 State of the Art

2.2 Own previous work

3. Study goals

4. Study population

4.1. Justification for the study of minors

4.2. Recruitment

4.3. Inclusion and exclusion criteria

4.4. Study medication

5. Study protocol and examination methods

5.1. Study design

5.2. Performance

5.3. Schedule

5.4. Examination methods

6. Criteria for interruption of the study

7. Risks and Adverse Reactions - Clinical and scientific assessment

8. Information regarding the statistical analysis

9. Clinical and scientific evaluation

10. Privacy policy and data security

11. Informed consent of the participants

12. Co-operations

13. Literature

14. Attachments

**1. SUMMARY**

The most common cause of homonymous visual field defects on the same side in both eyes (homonymous hemianopia) in children is pre- or perinatally acquired brain damage, followed by stroke, brain tumor, or a consequence of brain surgery, especially after epilepsy surgery. Hemianopia causes severe disability in everyday life, mainly in spatial orientation. There is a large deficit in specific diagnostic methods, which causes an urgent need for methods of vision rehabilitation programs in these children.

This study for the first time offers the chance to :
1. to examine structure-function relations (imaging vs. actual visual field loss);
2. to examine spontaneous compensatory eye movement strategies in children with hemianopia of different genesis and duration of disease.
3. to evaluate the efficacy and clinical application of a compensatory training method to improve orientation.
4. to examine preoperative and postoperative visual function after brain surgery due to epilepsy and tumors in order to make better predictions about surgery-induced damage in the future and to acquire information about adaptations to the cerebral visual field loss.

Due to the complexity of the primary disease and the serious damage to the children, the research project is not only of great scientific importance, but also with regard to the patient-oriented implementation, since it uses rehabilitation measures specifically with the ultimate goal of enabling the children to participate in life.

**2. Introduction**

**2.1 Background and state of the art**

Lesions of the visual pathway above the optic chiasm lead to visual field defects on the same side in both eyes (homonymous hemianopia). The visual field loss is limited to the vertical midline causing complete hemianopia, quadrantanopia or a small paracentral scotoma, depending on the location of the lesion. The most common cause of hemianopia in children is pre- or perinatally acquired brain damage, followed by childhood stroke, brain tumor, or surgery. Epilepsy and surgical interventions are of particular importance here. If patients with epilepsy do not adequately respond to drug therapy, surgery or removal (dissection) of the epileptogenic cerebral regions is possible in some cases. In the ideal case, this intervention leads to freedom from seizure. Yet there is the risk of leaving neurological deficits corresponding to the region of the brain damage. Visual field defects, especially hemianopia, are a frequent problem. In severe cases of epilepsy, the occurrence of such a visual field loss during surgery is even consciously taken into account.

Homonymous hemianopia causes a severe disability in everyday life, mainly regarding spatial orientation, so that patients bump into objects or people on the blind side, or have problems in wayfinding. Therefore, rehabilitation is particularly important regarding the hemianopic orientation disorder.

Previous studies have shown that adult patients can develop a spontaneous adaptation strategy by spontaneously making eye movements to the blind side to enlarge their field of gaze. This spontaneous compensation strategy can be improved by additional eye movement training (explorative saccadic training) (Kerkhoff et al., 1992, Zihl 1995, Pambakian et al., 2000, 2004, Roth et al., 2009), see below.

In contrast to extensive findings in adults, little is known so far, about compensation of damages to the visual pathway or the visual cortex in children - neither regarding europlastic processes in response to such damage nor regarding adaptive mechanisms and their targeted therapeutic-rehabilitative support. These questions are to be investigated in this research project, in order to provide the basis for a more effective treatment / rehabilitation of childhood visual field losses.

**2.2 Own previous work**

In the Vision Rehabilitation Research Unit and the associated Low Vision Clinic at the University Eye Hospital Tübingen, there is expertise of 20 years with visually impaired and blind patients. Since 1991, Prof. Trauzettel-Klosinski has been setting up this facility at the Tübingen University Eye Clinic. The close connection between service and research has proven to be highly successful, as it provides a good basis for patient-oriented research. In the last 15 years, about 30 research projects were financed by project funding (total funding volume: 3.921.475 €) and have been carried out in our research laboratory. In this way, we could assemble not only a competent team. but also a very good and solid collection of equipment.

Ophthalmologists and orthoptists collaborate as a team at the Low Vision Clinic and the Vision Rehabilitation Research Unit. Interdisciplinary cooperation, which is especially required for children with cerebral visual impairment, has been successfully practiced here for many years. There is also a special know-how in the examination and care of visually impaired children with additional disabilities, which was obtained by a 3-year research project funded by the Hildebrand and Palm Foundation for the evaluation of visual functions in children with multiple disabilities.

There is also special knowledge in the field of scientific analysis and rehabilitation of orientation and reading disorders (Trauzettel-Klosinski, see references). In the last few years, we have developed an evidence-based training method for adult patients with hemianopia after stroke (Roth et al 2009 a,b; Trauzettel-Klosinski, 2009). This study was awarded the Fürst-Donnersmark Research Award for Neurological Rehabilitation in 2009. This study showed that orientation and quality of life can be significantly and sustainably improved through targeted eye movement training (saccade training). The patients had to perform in a search task to train scanning eye movements to the blind side of their visual field. This leads to an increased awareness of the blind side with an attentional shift to their blind side and an expansion of their field of gaze). Although perimetry showed that the visual field defect, remained unchanged, the newly learned eye movement strategy allowed patients to better find persons and objects in real space.

The Department of Neurology and Developmental Neurology (Medical Director: Prof. Ingeborg Krageloh-Mann) of the University Clinic for Child and Adolescent Medicine has been studying early brain lesions for many years, regarding origin, effects and possibilities of compensation and reorganization. In addition to the motor domain (cerebral palsy), somatosensory, linguistic and cognitive aspects are also becoming increasingly important.

The main cooperation partner Prof. M. Staudt has been investigating questions about reorganization after early brain lesions in several research projects since 1999. One focus was on the multimodal detection of reorganized sensorimotor systems, using the methods of structural and functional MRI, diffusion tensor imaging with fiber tracking, transcranial magnetic stimulation and magneto-encephalography. Prof. Staudt is currently professor for "Neuroplasticity of the Child's Brain" at the Department of Neuro-Pediatrics and Developmental Neurology of the University Clinic for Child and Adolescent Medicine in Tübingen, and at the same time Chief Physician of the Department of Neurophysiology and Neurological Rehabilitation, Epilepsy Center for Children and Adolescents of the Schön -Klinik in Vogtareuth. This dual function provides ideal prerequisites for carrying out the research project, because the methodological and scientific expertise of the university (eye clinic and pediatric clinic) can be combined with the high number of suitable patients (both from epilepsy surgery and neurorehabilitation) of the Schön-Klinik Vogtareuth ,

The Schön-Klinik Vogtareuth is a highly specialized hospital for neuro-pediatrics, neurology and rehabilitation medicine as well as surgical disciplines. Neurology, neurosurgery and particularly the treatment of epilepsy in children and adolescents, the Schön-Klinik Vogtareuth has a trans-regional recruitment area. For example, 40-50 epilepsy surgeries are performed every year in children and adolescents. The special feature and the strength of the clinic is the combination of an epilepsy center for children and adolescents with a pediatric neurorehabilitation clinic, which enables holistic care "under one roof" as well as an intensive exchange between the two disciplines.

The second co-operation partner, PD. Dr. Wilke, leads the interdisciplinary work area "Experimental Pediatric Neuroimaging" at the Department of Neuropediatrics and Developmental Neurology of the University Clinic for Child and Adolescent Medicine. Based on extensive preliminary work, a project was recently started with the title "Pre-operative modern MR imaging in children". Our cooperation is to investigate visual function in children with brain tumors pre- and postoperatively, to determine structure-function relations (imaging versus actual visual field defect) and thus to improve the prediction of postoperative visual field losses. In addition, these children should also be examined regarding spontaneous adaptations to acute visual field losses. Rehabilitation by ways of saccade training should be provided.

**3. Aim of the study**The following aims are to be followed comparing children with pre- or perinatal acquired lesions (group A) and children with lesions acquired beyond the second year of life (group B)
1. Establishment of structure-function relationships between lesions of the visual pathway and/or visual cortex and of the actual visual field loss.

For comparable lesions we hypothesize: The earlier the lesion occurs, the less visual loss will there be.

2. Assessment of spontaneous adaptive mechanisms in children with hemianopia.

We hypothesize: The earlier the lesion occurs, the more pronounced and effective the compensation mechanisms are.

3. Evaluation of a child-friendly modification / optimization of the adult saccade training program for children with hemianopia (developed as part of the technical project up to the start of the study)

4. In children with acute hemianopia (in the context of surgical procedures for epilepsy, or after brain tumor surgery) the time course of the visual field loss and the development of compensation mechanisms are to be followed (group C).

**4. Study population** Group A: n = 30 children with pre- or perinatally acquired lesions of the visual pathway and / or visual cortex
 Group B: n = 10 children with later acquired lesions of the visual pathway and / or visual cortex (at least 2 years of age) - lasting at least one year.
 Group C: As many children as possible (about 4 per year) who develop iatrogenic acute and “planned” visual field defects during the course of epilepsy surgery (resections or disconnections of an occipital lobe, hemispherectomies). Additionally, children with acute hemianopia after brain tumor surgery (presumably 3-4 per year). The particularity of this study is due to the scarcity of the two diseases, which allows a precise evaluation of the spontaneous natural course of the cerebral visual field defect with the prospect of establishing a training method in the future.
 Group D: 10 healthy, age-matched control children will be examined to determine normal values for scanning eye movement behavior.

**4.1. Reason why the study must be carried out in children:**The most common cause of hemianopia in children is pre- or perinatally acquired brain damage, followed by childhood stroke, brain tumor, or surgery, especially after epilepsy-surgical procedures. Epilepsies are one the most common chronic diseases in childhood up to the age of ten years, with an incidence of 0.6 per thousand and a prevalence of 0.5 per thousand.

As there is little knowledge of structure-function relationship (MRI vs. actual visual field defect) in brain lesions acquired in early childhood and as there are few or no rehabilitation programs and methods available for these children, this study can be carried out only in children.

**4.2 Recruitment**The recruitment of the patients takes place mainly in the Schön-Klinik Vogtareuth.
The recruitment of the children with brain tumor is carried out in the University Children's Hospital Dept. III (Pediatric Neurology), Tübingen by PD Dr.Wilke, our direct cooperation partner. The surgery is performed in the Neurosurgery Department of the University Hospital Tübingen.

The recruitment of the control children takes place from the circle of friends of the employees of the Visual Rehabilitation Research Unit. Special attention will be paid to the aspect of voluntary participation.

**4.3. Inclusion and exclusion criteria**
Inclusion criteria
 - Age 6-18 years, preferably patients aged 10 or above
 - Presence of neuro-pediatric findings
 - Presence of MRI findings
 - Informed consent from at least one parent
Exclusion criteria
 - Lack of cooperation

**4.4. Study medication**No drugs are tested in this study. Dilatation of the pupil and cycloplegia may be necessary in some children (see chapter 7).

**5. Course of study and examination methods
5.1 Study design**The proposed research project is a prospective, clinical diagnostics and intervention study that is closely linked to a laboratory research project for the development of examination and rehabilitation methods in children with hemianopia (see flow chart). The technical preparations are already underway. All examinations and activities will be carried out through intense cooperation between the Vision Rehabilitation Research Unit Centre for Ophthalmology at the University of Tuebingen, The Department of Neuropediatrics, Developmental Neurology Neurological Rehabilitation and Epilepsy (Children and Adolescents) at the University Hospital for Children and Adolescents Tuebingen, and the Department of Neuropediatrics, Neurological Rehabilitation and Epilepsy (Children and Adolescents) of the Schön-Klinik Vogtareuth. The advantage of such an interdisciplinary research project is the close coordination of individual, high-quality services (ophthalmology and neuro-pediatrics), so that the children receive optimal diagnostic and rehabilitation measures.

**5.2 Implementation**

The examinations are carried out in the Vision Rehabilitation Research Unit, Centre for Ophthalmology at the University of Tuebingen in the ViTa Building Osianderstr. 5.

**5.3 Schedule**The project consists of a clinical part and a technical part. The latter supports the study technically and has already carried out preliminary work with a lead of 7 months. This allows the clinical project to begin at the same time as the pilot study (see graph).

**5.4. Methods of Examination**
All examinations are performed on an outpatient basis. The complete examination protocol takes about 3 hours per patient with additional breaks.
**5.4.1. Determination of visual acuity for distance and near at standardized illumination** using different vision tests depending on age and clinical severity of the disease, as well as the ability to cooperate. In addition, objective eye refraction tests will be applied, including retinoscopy and refractometry.

**5.4.2.** **Contrast sensitivity tests are performed using Lea numbers or symbols at 10% contrast.**
**5.4.3.** **Orthoptic status:** fixation, eye position, motility, binocular vision, eye dominance.

**5.4.4.** **Visual field examination:** with regard to everyday activities such as near work, reading and orientation in general, the central 30 ° visual field is examined, if possible with the Tübingen Manual Perimeter (kinetic). Severely disabled children are not able to cooperate for conventional perimetry. In these children, a tangent-screen campimetry is carried out, where white stimuli are presented manually on a black background on a wall. Even less cooperation is required by the LED campimetry, which was developed in our research group, in which eye movements towards the stimulus are allowed. In the 30 ° visual field, light stimuli are presented by white light-emitting diodes. More specifically, this is a measurement of the field of gaze. The test uses a reflexive response and does not depend on subjective information from the patient. All other examination methods have already been used in previous studies with positive ethics committee approval (see page 1), except point 6 (see there). All examinations are carried out only by qualified employees. All devices are used and maintained only for their intended purpose and are kept in good condition.

**5.4.5.** **Clinical-morphological examination of the anterior and posterior segments of the eye** with an accurate assessment of the ophthalmological findings.

**5.4.6.** **Eye movements** while viewing a natural scene on the screen as well as during search tasks using an infrared eye tracker (Saccadometer, Jazz novo, Ober). The measuring unit is located on the nasal root and illuminates the limbus tangentially. It is attached to the head with an elastic band. The advantage of this system, especially for children, is the fact that it is not placed directly in front of the eyes. At initial examination (baseline), spontaneous adaptation strategies, after the training the potential improvements are evaluated. An in-house certification is currently underway. The certificate will be submitted.

**5.4.7. Saccade training** with a software program suitable for children that consists of a search task on the screen: The child receives instructions at our site and can then practice at home on his own computer independently: 2x15 min / day on 5 days / week for 6 weeks. If a personal computer is not available, the child receives a laptop computer from us.

**6. Termination criteria**

Participation in the study can be terminated by the patient without telling the reason at any time. In addition, mandatory termination criteria are strong exertion or fatigue on the part of the patient.

**7. Risks and side effects**

The ophthalmological examinations carried out within the framework of the study correspond to those of a routine examination with regard to risks and possible side effects. Most of the examination methods are standardized and have already been routinely used in the clinic.
For the examination of the fundus of the eye, pupil dilation with a combined parasympatholytic and sympathomimetic drug can be necessary. In order to enlarge the pupil, we use the parasympatholytic drug tropicamide topically, which is also used in routine ophthalmological examinations. The risk of acute glaucoma due to pupillary dilation is hardly ever occurs in children.
For objective determination of refraction, cycloplegia is sometimes necessary in younger children. For this purpose, we give eye drops containing the active ingredient cyclopentolate hydrochloride (cyclopentolate 1%, or 0.5%), generally 2-3 times at 10 minute intervals. Before, we ask the neuropediatrists for a brief written recommendation, which eye drops we may use.

The pupillary dilation lasts for about 3-4 hours. During this time, there may be an increased sensitivity to light and temporally reduced near vision.
The LED campimetry that was developed in our research group, only uses light-emitting diodes with EU safety markings (already used in earlier studies).
Current standard computers are used for the training and search tasks, which, according to the applicable regulations, are low-radiation computers (already used in previous studies).
The infrared eye tracker used here is particularly well suited for children, since it is only attached with a headband and the measuring unit does not sit directly in front of the eyes. We have already used other infrared eye trackers in previous studies.

**8. Clinical and scientific impact**
The study is not only of innovative scientific importance but also very patient-oriented. The training results is a benefit for the children and can be used to complete everyday tasks.

**9. Information on statistical evaluations**

The findings of the ophthalmological examinations are collected separately from the neuropediatric and MRI examinations and entered into a database. Afterwards they can be compared with the clinical-neurological and MRI -structural findings on the basis of the pseudonymized assignment.
The focus will be on the correlation between ophthalmological, neuropediatric and MRI findings.

**10. Privacy Policy and Data Security**

Data are collected on questionnaires, examination sheets, or in computer files. Data are stored in pseudo-randomized form immediately after acquisition. For this purpose, a code is assigned and a list is made that allows retrieving the data.

The coding documents and the coding list are stored together with the declarations of consent in a locked cabinet, to which only the investigators have access. It is made accessible to third parties only in the cases specified in the declaration of consent to data protection. Data are stored for 10 years.

**11. Informed consent by the participants**
The investigator informs each subject/patient and at least one parent in detail about the examination before their consent. All subjects / patients and their parents are explicitly informed that they can terminate their participation or the participation of their child in the study at any time and without giving reasons. Only after this procedure, the investigators and their parents give their written informed consent to the examinations.

In case a deficit occurs, parents of children will be informed during the post-surgical examination, about the possible participation in a follow-up study (saccade training), where rehabilitation and reorganization will be investigated together with Professor Staudt.

Remuneration: After completion of the last examination, the children receive a book voucher worth € 25.

**12. Cooperations (for details see page 1)**

Schön-Klinik Vogtareuth

Prof. Martin Staudt MD, Chief Physician of the Department of Neuropediatrics and Neurological Rehabilitation, Epilepsy Center for Children and Adolescents, Schön-Klinik Vogtareuth

Department of Neuropathology, Tübingen

PD Marko Wilke MD: Cooperation partner in the investigation of children with brain tumors

Prof.Martin Staudt MD, Foundation Professor for "Neuroplasticity of the child`s brain"

Tübingen, den 24.07.2014

Prof. Dr. med. Susanne Trauzettel-Klosinski

**13. References**

References regarding the topic

**References of other authors**

- Ahmed M, Dutton GN (1996). Cognitive visual dysfunction in a child with cerebral damage. Developmental Medicine and Child Neurology 38: 736-743
- Baker-Nobles L, Rutherford A (1995). Understanding cortical visual impairment in children. American Journal of occupational Therapy 49: 899-903
- Barkovich AJ, (1992). MR and CT evaluation of the profound neonatal and infantile asphyxia. Am J Neuroradiol 13: 959-972
- Buonomano DV, Merzenich MM (1998) Cortical plasticity: From synapses to maps. Avv Rev Neurosci 2: 149-186
- Dutton GN, Ballantyne J, Boyd G. Bradnam M, Day R, McCulloch D, Mackie R, Phillips S, Saunders K (1996). Cortical visual dysfunction in children: a clinical study. Eye 10: 302-309
- Dutton GN, Jacobson LK (2001). Cerebral visual impairment in children. Semin Neonatol. Dec; 6 (6):477-85. Review
- Good WV, Jan JE, Burden SK, Skoczenski A, Candy R. Recent advances in cortical visual impairment (2001). Dev Med Child Neurol. Jan;43(1): 56-60. Review
- Good WV, Jan JE, deSa L, Barkovich KAJ, Groenveld M, Hoyt CS (1994). Cortical visual impairment in children: a major review. Survey of Ophthalmology 88: 351-364
- Groenveld M, Jan JE, Leader E (1990). Observations on the habilitation of children with cortical visual impairment. Journal of Visual Impairment and Blindness 84: 11-15
- Guzzetta A, Mercuri E, Cioni G (2001). Visual disorders in children with brain lesions: 2. Visual impairment associated with cerebral palsy. Eur J Paediatr Neurol. 5(3): 115-9, Review
- Guzzetta A, D'Acunto G, Rose S, Tinelli F, Boyd R, Cioni G. Plasticity of the visual system after early brain damage. Dev Med Child Neurol. 2010 Oct;52(10):891-900. doi: 10.1111/j.1469-8749.2010.03710.x. Epub 2010 Jun 15. Review.
- Guzzetta A, Fiori S, Scelfo D, Conti E, Bancale A. Reorganization of visual fields after periventricular haemorrhagic infarction: potentials and limitations. Dev Med Child Neurol. 2013 Nov;55 Suppl 4:23-6. doi: 10.1111/dmcn.12302. Review.
- Hoyt CS (2003). Visual function in the brain-damaged child. Eye. Apr; 17(3): 369-84, Review
- Hoyt CS (2007). Brain injury and the eye. Eye 21: 1285-1289
- Jacobson L, Flodmark O, Martin L. Visual field defects in prematurely born patients with white matter damage of immaturity: a multiple-case study. Acta Ophthalmol Scand. 2006 Jun;84(3):357-62.
- Jacobson L, Rydberg A, Eliasson AC, Kits A, Flodmark O. Visual field function in school-aged children with spastic unilateral cerebral palsy related to different patterns of brain damage. Dev Med Child Neurol. 2010 Aug;52(8):e184-7. doi: 10.1111/j.1469-8749.2010.03650.x. Epub 2010 Apr 30
- Kerkhoff, G., Münßinger, U. Haaf, E. Eberle-Strauss, G., Stögerer, E. (1992): Rehabilitation of homonymous scotomata in patients with postgeniculate damage of the visual system: saccadic compensation training. Restor neurol Neurosci 4: 245-254
- Kommerell, G., Lieb, B., Münßinger, U.(1999): Rehabilitation bei homonymer Hemianopsie. Z prakt Augenheilkunde 20: 344-352
- Pambakian, A.L., Mannan, S.K., Hodgson, T.L., Kennard, C. (2004) Saccadic visual search training: a treatment for patients with homonymous hemianopia. J Neurol Neurosurg Psychiatry 75, 1443–1448
- Pambakian, A.L., Wooding, D.S., Patel, N., Morland, A.B., Kennard, C.,
  Mannan, S.K. (2000) Scanning the visual world: a study of patients with homonymous hemianopia. J Neurol Neurosurg Psychiatry 69, 751–759
- Tinelli F, Guzzetta A, Bertini C, Ricci D, Mercuri E, Ladavas E, Cioni G. Greater sparing of visual search abilities in children after congenital rather than acquired focal brain damage. Neurorehabil Neural Repair. 2011 Oct;25(8):721-8. doi: 10.1177/1545968311407780. Epub 2011 Jun 6.
- Zihl, J.(1995): Visual scanning behaviour in patients with homonymous hemianopia. Neuropsychologia 33: 287-303

**Own publications regarding the topic**

- Trauzettel - Klosinski S (1997) Eccentric fixation in hemianopic field defects - a valuable strategy to improve reading ability and an indication for cortical plasticity. Neuro - Ophthalmol 18: 117-131
- Trauzettel - Klosinski S, Brendler K (1998) Eye movements in reading with hemianopic field defects: the significance of clinical parameters. Graefe`s Arch Clin Exp Ophthalmol 236: 91-102
- Trauzettel - Klosinski S, Reinhard J (1998) The vertical field border in human hemianopia and its significance for fixation behavior and reading. Invest Ophthalmol Vis Sci 39. 2177-2186
- *Atwell Award und Fortune Poster-Preis - ???*
- Trauzettel-Klosinski S (2004) Rehabilitation bei homonymer Hemianopsie - ein Überblick. Z prakt Augenheilk 25: 298-304
- Reinhard J, Schreiber A, Vonthein R, Schiefer U, Trauzettel-Klosinski S (2004) Visuelles Restitutionstraining bei homonymer Hemianopsie. Z prakt Augenheilk 25: 305-312
- Reinhard J, Schreiber A, Schiefer U, Sabel BA, Kasten E, Kenkel S, Vonthein R, Trauzettel-Klosinski S (2005) Does visual restitution training change absolute homonymous scotoma? Brit J Ophthalmol 89: 30-35
- Roth T, Sokolov AN, Messias A, Roth P, Weller M, Trauzettel-Klosinski S. (2009) Comparing explorative saccade and flicker training in hemianopia: a randomized controlled study. Neurology 72(4): 324-331.
- *Fürst Donnersmarck Forschungspreis für Neurorehabilitation*
- Roth T, Sokolov A, Messias A, Roth P, Weller M, Trauzettel-Klosinski S (2009) Sakkadentraining verbessert visuelle Exploration bei Hemianopsie – Eine randomisierte kontrollierte Studie. Z prakt Augenheilk 30:403-410
- Trauzettel-Klosinski S (2009) Rehabilitation bei Sehbahnschäden. Klin Monatsbl Augenheilk 226: 897-907
- Trauzettel-Klosinski S (2010) Rehabilitation for Visual Disorders. J Neuro-Ophthalmol 30: 73-84
- Trauzettel-Klosinski S (2011) Zeitgemäße Möglichkeiten visueller Rehabilitation -
- Up-to date Options for Visual Rehabilitation. Dtsch Aerztebl 108, 51/52, 871-878
- Cordey A, Trauzettel-Klosinski S (2012) Exploratives Sakkadentraining – Kompensation von homonymen Gesichtsfelddefekten. Orthoptik - Pleoptik 35: 19-28
- Trauzettel-Klosinski S (2012) Visuelles Rehabilitationstraining bei homonymen Gesichtsfeldausfällen. Ophthalmologe, 109:496 - 500 / DOI 10.1007/s00347-012-2571-6
- Trauzettel-Klosinski S, Dietz K and the IReST Study Group (2012) Standardized Assessment of Reading Performance: The new International Standardized Reading Texts IReST. Invest Ophthalmol Vis Sci 53:5452-5461
- Reinhard J, Damm I, Ivanov IV, Trauzettel-Klosinski S (2014) Eye movements during saccadic and fixation tasks in patients with hemianopia. J Neuro-Ophthalmol (in print

**References of the cooperation partners regarding the topic**

- Pascoal T, Paglioli E, Palmini A, Menezes R, Staudt M: Immediate improvement of motor function after epilepsy surgery in congenital hemiparesis. Epilepsia. 2013; 54(8): e109-111
- van der Kolk N, Boshuisen K, van Empelen R, Koudijs S, Staudt M, van Rijen P, van Nieuwenhuizen O, Braun K: Etiology-specific differences in motor function after hemispherectomy. Epilepsy Res 2013 Feb;103(2-3):221-30
- Zsoter A, Pieper T, Kudernatsch M, Wilke M, Staudt M: Predicting hand function after hemispherotomy: TMS versus fMRI in hemispheric polymicrogyria. Epilepsia 2012, 53(6):e98-e101
- Juenger H, DeHaan B, Krägeloh-Mann I, Staudt M, Karnath HO: Early determination of somatosensory cortex in the human brain. Cerebr Cortex 2011;21(8):1827-31
  - *Desitin-Jungforscherpreis der Deutschen Gesellschaft für Neuropädiatrie 2011 –*
- Staudt M: Reorganization after pre- and perinatal brain lesions. Journal of Anatomy; Special Edition: Development of the Neocortex. 217(4):469-474 (2010) [invited review]
- Wilke M, Staudt M, Juenger H, Grodd W, Braun C, Krägeloh-Mann I: Somatosensory system in two types of motor reorganization in congenital hemiparesis: topography & function. Hum Brain Map, 2009; 30: 776-788
- Staudt M, Ticini LF, Grodd W, Krägeloh-Mann I: Functional topography of early periventricular brain lesions in relation to cytoarchitectonic probabilistic maps. Brain Lang 2008; 106:177-83.
- Staudt M: (Re-)organization of the developing human brain following periventricular white matter lesions. Neurosci Biobehav R 31 (2007) 1150–1156
- Staudt M, Braun C, Gerloff C, Erb M, Grodd W, Krägeloh-Mann I: Developing somatosensory projections bypass periventricular brain lesions. Neurology 2006;67: 522-525
- Staudt M, Erb M, Braun C, Gerloff C, Grodd G, Krägeloh-Mann I: Extensive perlesional connectivity in congenital hemiparesis. Neurology 66: 771 (2006)
- Staudt M, Gerloff C, Grodd W, Holthausen H, Niemann G, Krägeloh-Mann I: Reorganization in congenital hemiparesis acquired at different gestational ages. Ann Neurol, 56 (2004): 854-863
- *– Gayle G. Arnold Award for Excellence in the Care of Children with Cerebral Palsy
   der American Academy for Cerebral Palsy and Developmental Medicine AACPDM–*
- Staudt M, Grodd W, Gerloff C, Erb M, Stitz J, Krägeloh-Mann I: Two types of ipsilateral reorganization in congenital hemiparesis: a TMS and fMRI study. Brain 125 (2002):2222-37
  - *Desitin-Jungforscherpreis der Deutschen Gesellschaft für Neuropädiatrie 2003 –*
- Staudt M, Grodd W, Niemann G, Wildgruber D, Erb M, Krägeloh-Mann I: Early left periventricular brain lesions induce right hemispheric organization of speech. Neurology 57 (2001): 122-125
  - *Alois-Kornmüller-Preis der Deutschen Gesellschaft für Klinische Neurophysiologie –*
- Wilke M, Pieper T, Lindner K, Dushe T, Staudt M, Grodd W, Holthausen H, Krägeloh-Mann I (2011): Clinical functional MRI of the language domain in children with epilepsy. Hum Brain Mapp 32: 1882-1893
- Zsoter A, Staudt M, Wilke M (2012). Identification of successful clinical fMRI sessions in children: an objective approach. Neuropediatrics 43: 249-57

14. Appendices

Brief information on the study for children from Vogtareuth
Brief information on the study for children with brain tumors from Tübingen
Detailed information on the study for children from Vogtareuth
Detailed information on the study for children with brain tumors from Tübingen
Declaration of consent of patients / parents
